# Frozen bean curd-inspired xenogeneic acellular dermal matrix with triple pretreatment approach of freeze–thaw, laser drilling and ADSCs pre-culture for promoting early vascularization and integration

**DOI:** 10.1093/rb/rbac053

**Published:** 2022-08-04

**Authors:** Xing Huang, Zhu Zhu, Lin Lu, Rui Jin, Di Sun, Xusong Luo

**Affiliations:** Department of Plastic and Reconstructive Surgery, Shanghai 9th People's Hospital, Shanghai Jiao Tong University School of Medicine, Shanghai 200011, PR China; Shanghai Key Lab of Tissue Engineering, Shanghai 9th People's Hospital, Shanghai Jiao Tong University School of Medicine, Shanghai 200011, PR China; Department of Plastic and Reconstructive Surgery, Shanghai 9th People's Hospital, Shanghai Jiao Tong University School of Medicine, Shanghai 200011, PR China; Shanghai Key Lab of Tissue Engineering, Shanghai 9th People's Hospital, Shanghai Jiao Tong University School of Medicine, Shanghai 200011, PR China; Department of Plastic and Reconstructive Surgery, Shanghai 9th People's Hospital, Shanghai Jiao Tong University School of Medicine, Shanghai 200011, PR China; Department of Plastic and Reconstructive Surgery, Shanghai 9th People's Hospital, Shanghai Jiao Tong University School of Medicine, Shanghai 200011, PR China; Department of Plastic and Reconstructive Surgery, Shanghai 9th People's Hospital, Shanghai Jiao Tong University School of Medicine, Shanghai 200011, PR China; Department of Plastic and Reconstructive Surgery, Shanghai 9th People's Hospital, Shanghai Jiao Tong University School of Medicine, Shanghai 200011, PR China

**Keywords:** freeze–thaw, xenogeneic acellular dermal matrix, vascularization, early integration

## Abstract

Xenogeneic acellular dermal matrix (ADM) is widely used in clinical practice given its good biocompatibility and biomechanical properties. Yet, its dense structure remains a hindrance. Incorporation of laser drilling and pre-culture with Adipose-derived stem cells (ADSCs) have been attempted to promote early vascularization and integration, but the results were not ideal. Inspired by the manufacturing procedure of frozen bean curd, we proposed a freeze–thaw treatment to enhance the porosity of ADM. We found that the ADM treated with −80°C 3R+−30°C 3R had the largest disorder of stratified plane arrangement (deviation angle 28.6%) and the largest porosity (96%), making it an optimal approach. Human umbilical vein endothelial cells on freeze–thaw treated ADM demonstrated increased expression in Tie-2 and CD105 genes, proliferation, and tube formation in vitro compared with those on ADM. Combining freeze–thaw with laser drilling and pre-culture with ADSCs, such tri-treatment improved the gene expression of pro-angiogenic factors including IGF-1, EGF and vascular endothelial growth factor, promoted tube formation, increased cell infiltration and accelerated vascularization soon after implantation. Overall, freeze–thaw is an effective method for optimizing the internal structure of ADM, and tri-treatments may yield clinical significance by promoting early cell infiltration, vascularization and integration with surrounding tissues.

## Graphical Abstract

**Figure:**
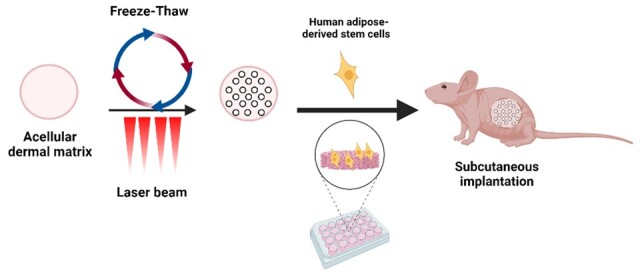

## Introduction

Acellular dermal matrix (ADM) is a natural 3D biomaterial mainly composed of collagen fibers without antigens like cells and glands [1]. Due to its biocompatibility, mechanical properties, abundant source and immunological inertia, it has been widely used in clinical practice [2, 3], involving procedures of deep wound coverage, breast reconstruction, abdominal wall reconstruction and rhinoplasty. These properties have attracted the attention of researchers in the field of tissue engineering [4, 5]. Additionally, it has been applied as an injectable tissue filler in the field of medical esthetics in the past few years given its good shaping ability and is expected to be an excellent tissue filler ranked directly under hyaluronic acid and collagen [6]. However, derived from the dermis papillary layer, the collagen fibers are arranged in an orderly and dense manner thus lacking sufficient micropores [4]. This poses a hindrance for cell infiltration and angiogenesis or vascularization, stopping ADM from becoming the ideal scaffold for early integration. Clinical studies have found that patients with a history of radiotherapy were more prone to complications, such as seroma and implant loss after breast reconstruction when Porcine ADM (PADM) was used due to delayed or inhibited vascularization and cell infiltration caused by radio [7]. Abdominal wall reconstruction with PADM also reported up to 35% complication rate including hernia recurrence and abdominal wall skin necrosis [8–10]. Therefore, solving the difficulties of early vascularization and integration after ADM implantation caused by dense structure would decrease the occurrence of ADM-related complications and improve the quality of patients’ postoperative life.

Adipose-derived stem cells (ADSCs) seeded on specific tissues would promote local vascularization by differentiating to adult cells or secreting active factors [11–13]. Our previous studies found that the combined treatment of laser micropore technique and ADSCs pre-culture promoted the growth of dermis flap constructed from treated ADM and fat tissue [14]. Pores drilled by laser significantly increased the contact area with host tissue, while ADSCs pre-culture formed a pro-angiogenic microenvironment by paracrine. However, the laser micropore technique merely changed local permeability, and the unperforated zone remained dense. In addition, the size, connectivity and geometry of pores are known to influence a range of cell behaviors, including cell proliferation, migration and infiltration, and extracellular matrix deposition [15]. Therefore, there is value to a new method that increases the void fraction of ADM by increasing the internal micropore or pore diameter.

**Figure 1.**
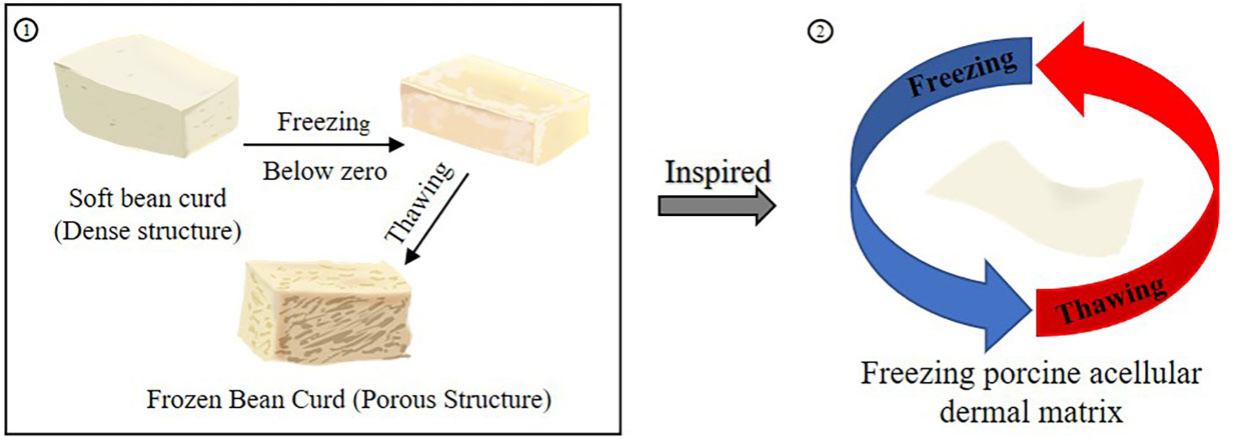
Schematical representation of FADM. (1) The procedure of frozen bean curd. Soft bean curd was frozen below zero and thawed at room temperature to obtain frozen bean curd. (2) Freeze–thaw treatment for PADM with different temperature gradients and cycles.

Frozen bean curd is a common food in East Asia. Soft bean curd (a gel composed of soybean particles and water) is frozen below zero into blocks and then thawed at room temperature to obtain frozen bean curd with multiple pores. Inspired by its manufacturing process, we proposed the freeze–thaw treatment to ADM (Fig. 1). The ice crystals formed during the cooling process are influenced by the temperature gradient. The greater the temperature gradient, the smaller crystals formed [16, 17]. Furthermore, the cycles of freeze–thaw also affect the connectivity of internal pores [18–20]. It has been found that three cycles of freezing (−20°C) and water bath melting (37°C) could increase the interconnectivity among pores of a porcine acellular aortic stent. Temperatures of −20°C, −80°C and −196°C were applied resulting in cryogels of different total porosity and porous interconnectivity [21]. Therefore, it is feasible to introduce porosity through freeze–thaw. Yet so far, no report regarding the ADM reconstruction by freeze–thaw has been published. In addition, the effect of freeze–thaw treatment on vascularization of ADM has not been reported. Thus, we investigated whether porous ADM with high connectivity could be obtained through the appropriate freezing rate and freeze–thaw cycles.

Herein, inspired by frozen bean curd and based on previous studies’ founding, we propose a triple *in vitro* pretreatment combing freeze–thaw, laser drilling and hADSCs pre-culture to construct an ADM into a 3D porous scaffold seeded with hADSCs. Experiments were conducted *in vitro* to verify the pro-angiogenic effect of freeze–thaw treatment on ADM through observing the HUVECs’ behaviors on PADM and freeze–thaw treated ADM (FADM). Furthermore, pre-implanted microenvironment, cell infiltration and vascularization that play significant roles in the early integration of the ADM treated with triple measures were assessed *in vitro* and *in vivo*.

## Materials and methods

### Material preparation

PADM was purchased from Jiangsu Unitrμmp Bio-medical Technology Co. (Jiangsu, China) and cut to sizes of 5 × 5 cm slices.

#### Freeze–thaw treatment

The slices soaked in phosphate-buffered saline (PBS) were frozen at temperatures below zero temperature (−80°C, −30°C or liquid nitrogen/−196°C) for 1 h, water-bathed at 37°C over half an hour until thawed completely, and repeatedly freeze-thawed for 3, 4, 6 or 8 cycles in total. The FADM was rinsed with PBS and stored at −80°C until use. The details of temperature, duration and cycle are shown in Table 1.

**Table 1.** The different freeze–thaw treatments of PADM

No.	Freezing temperature (°C)	Duration (h)	Thawing temperature (°C)	Duration (h)	Cycles	Freezing temperature (°C)	Duration	Thawing temperature (°C)	Duration (h)	Cycles
1	/	/	/	/	/	/	/	/	/	/
2	−80	1	37	0.5	2	−30	1	37	0.5	2
3	−80	1	37	0.5	3	−30	1	37	0.5	3
4	−80	1	37	0.5	4	−30	1	37	0.5	4
5	−80	1	37	0.5	8					
6	−30	1	37	0.5	2	−80	1	37	0.5	2
7	−30	1	37	0.5	3	−80	1	37	0.5	3
8	−30	1	37	0.5	4	−80	1	37	0.5	4
9	−30	1	37	0.5	8					
10	−196	1	37	1	3					

sample1 was without any freeze-thaw treatment.

#### Laser micropore technique treatment

The microporous slices were prepared using a 15w UV picosecond Laser System Micro Material Processing (DCT Co., Ltd, China). Key parameters included 1000 mm/s speed, 500k frequency, 1-mm spot diameter, 1-mm pitch row and 10–12 repeats which were adjusted according to the thickness of the material to achieve maximum vertical penetration and minimum heat damage. The laser-treated ADM (LADM) was rinsed in an ultrasonic cleaner to remove degenerated tissues inside the micropores. Then LPADM was stored at −80°C until use.

#### Combined treatment

The slices were fabricated using the laser micropore technique first. Then the LADM was further frozen at the optimal setting that yielded the most porous structure. The freezing-laser-treated ADM (FLAMD) was rinsed in an ultrasonic cleaner and stored at −80°C until use.

All above materials were all cut to match the size of the wells of a 24-well plate and disinfected using ethylene oxide after freeze-drying.

### Scanning electron microscopy and histology of PADM and FADM

The samples were dried using the freezing-drier (YB-FD-1, SHYB Co., Ltd, China). The surface morphology of the dermal surface and cross-section after cutting up with liquid nitrogen were scanned by scanning electron microscopy (SEM) (ZEISS Gemini 300, Germany).

PADM and FADM were fixed with 4% paraformaldehyde, embedded in paraffin, then cut into sections at a thickness of 5 μm. Sections were stained with hematoxylin and eosin (H&E) and observed under a light microscope (Nikon, Tokyo, Japan).

### Porosity of PADM and FADM

The PADM and the FADM were prepared in sizes of 5 × 5 cm and then freeze-dried. Slices of 1 × 1 cm in size were clipped in the center of the lyophilized samples. The cleaned and dried samples were first weighed (*W*_0_). These were then immersed in anhydrous ethanol at room temperature and the bubbles in them were removed through vacuum pumping. Fully saturated samples were then removed from the liquid, immediately rolled on a filter paper to remove the surface film of anhydrous ethanol, and weighted (*W*_e_). The volume of samples (*V*_s_) was calculated by measuring their length, width and thickness. Pore volume (*V*_p_) and porosity were calculated using the following equations:

$$\begin{array}{c} V_{p}=\frac{\left(W_{e}-W_{0}\right)}{\rho }\\ \text{Porosity}=\frac{V_{p}}{V_{s}}\times 100\% \end{array},$$

where ρ is the density of anhydrous ethanol.

### *In vitro* experiments of human umbilical vein endothelial cells on PADM and FADM

Human umbilical vein endothelial cells (HUVECs) were obtained from the cell bank of the Shanghai Institute of Cell Biology, Chinese Academy of Sciences (Shanghai, China). HUVECs were incubated in a low glucose medium (Gibco, USA) supplemented with 10% fetal bovine serum (CellMax, China) and 1% streptomycin–penici1lin–amphotericin B solution (Gibco), and incubated at 37°C in a humidified atmosphere containing 5% CO_2_.

#### Cell proliferation and tube formation assay

HUVECs (1 × 10^4^ cells/well) were seeded on the PADM and FADM. After 1, 4 and 7 days of incubation, cell proliferation was assessed with Cell Counting Kit (CCK)-8 (Dojindo, Japan) according to the manufacturer’s instructions. After incubating at 37°C for 3 h, the absorbance of samples was measured at a wavelength of 450 nm using an MP reader (Thermo, USA).

For the tube formation assay, HUVECs (5000/well) were seeded into Matrigel-coated (Corning, USA) PADM and FADM in a 96-well plate and treated with serum-free culture medium [22], and then examined by bright-field microscopy at 6 h.

#### Morphology and viability of HUVECs

HUVECs (5 × 10^4^) seeded on PADM and FADM and cultured for 2 days were examined by SEM. The samples were fixed overnight at 4°C in 2.5% glutaraldehyde and rinsed with PBS three times (15 min each time). Then the samples were dehydrated through a graded series of ethanol (30–100%, V/V) and dried by CO_2_ critical point dryer. Once dried, the samples were observed under an SEM (SU8010, HITACHI, Japan).

The viability of HUVECs (1 × 10^4^) seeded on PADM and FADM was measured with the Live/Dead cell viability assay (Beyotime Biotechnology, China) according to the manufacturer’s instruction on Days 1 and 3.

#### Expression of endothelial marker genes: quantitative real-time PCR

HUVECs (1 × 10^5^) were seeded on circular PADM and FADM and cultured in a complete low glucose medium for 48 h. After cells were detached from the materials by trypsin (Gibco), total RNA was extracted and reverse transcribed into cDNA with PrimeScript RT Master Mix using RNA purification kit and Reverse Transcription kit (with DNase) (EZBioscience, USA). Gene expression levels of endothelial markers of CD105 (Endoglin/ENG), and Tie-2 (TEK receptor tyrosine kinase/TEK) [23] were normalized to that of glyceraldehyde 3-phosphate dehydrogenase (GAPDH) and quantified with the comparative Ct method. The primers synthesized by Sangon Biotech Co. (Shanghai, China) are mentioned in Table 2.

**Table 2.** Primer sequence for qPCR

Cell	Gene	Forward primer	Reverse primer
HUVEC	GAPDH	CAGGAGGCATTGCTGATGAT	GAAGGCTGGGGCTCATTT
	ENG	CTTCATGCGCTTGAACATCATC	GAGTAGATGTACCAGAGTGCAG
	TEK	CGTGATTGACACTGGACATAAC	GAGTTCATATTCTGTCCGAGGT
hADSC	GAPDH	CAGGAGGCATTGCTGATGAT	GAAGGCTGGGGCTCATTT
	VEGF	ATCGAGTACATCTTCAAGCCAT	GTGAGGTTTGATCCGCATAATC
	EGF	GAAGCATTGGACAAGTATGCAT	CAGCTTCTGAGTCCTGTAGTAG
	IGF-1	AAAAATCAGCAGTCTTCCAACC	CCTGTGGGCTTGTTGAAATAAA

### Isolation and culture of hADSCs

Human ADSCs (hADSCs) were isolated from five healthy female patients undergoing abdominal liposuction surgeries between May 2021 and November 2021. Written informed consent was provided by all patients. The average age of patients was 28 years (range, 22–32years). An equal volume of 0.1% (W/V) collagenase NB4 (Serva, Heidelberg, Germany) in serum-free low-glucose Dulbecco’s Modified Eagle’s Medium (DMEM, Gibco) was added to washed adipose tissue, and the mixture was digested at 37°C for 2 h to a homogeneous state. The lipoaspirate was centrifuged at 1200 rpm for 8 min at 37°C. The cells were concentrated and then resuspended in DMEM containing 10% fetal bovine serum (CellMax, China) and 1% streptomycin–penici1lin–amphotericin B solution (Gibco), and seeded into tissue culture flasks. The cells were incubated at 37°C contained 5% CO_2_ and cultured to 90% confluence before passaging. hADSCs of passages 3–4 were used for the experiments.

### Identification of hADSCs

hADSCs were tested for their ability to undergo tri-lineage differentiation into adipocytes, osteoblasts, and chondrocytes. Passage 3 hADSCs at a density of 2 × 10^5^ cells/ml were seeded into six-well plates, induced for 3 weeks with either adipogenic (Cyagen, China), osteogenic (Cyagen) differentiation media, and induced for 4 weeks with chondrogenic (Cyagen) differentiation media according to manufactures’ instructions. The cells were then fixed in 4% paraformaldehyde and stained with either Oil Red O (Beyotime, China), Alizarin red (Beyotime) or Toluidine blue (Beyotime) according to the standard procedure.

The surface markers of hADSCs were detected by flow cytometry. hADSCs (Passage 3) were harvested and incubated with fluorescein isothiocyanate or phycoerythrin-conjugated antibodies against CD90, CD44, CD105, CD34, CD45, CD106 (Santa Cruz Biotechnology, Inc., Santa Cruz, CA, USA) at 37°C for 30 min in the dark, washed and resuspended in PBS and detected by flow cytometry (BD Biosciences, San Jose, CA, USA).

### Adhesion of hADSCs on PADM, FADM, LADM and FLADM

hADSCs (1 × 10^5^) were seeded on PADM, FADM, LADM and FLADM and cultured for 2 days. The adhesion of hADSCs on materials was evaluated with Live/Dead cell viability assay (Beyotime Biotechnology) and observed under a laser scanning confocal microscope (Leica, German).

### Toxicity and cytocompatibility of PADM, FADM, LADM and FLADM

The toxicity of PADM, FADM, LADM and FLADM was indirectly evaluated, extracts from the above materials were prepared by incubation in 1-ml DMEM containing 10% FBS and 1% streptomycin–penici1lin–amphotericin B solution (Gibco) for complete immersion in a 24-well plate for 72 h. The extract was collected and passed through a filter (Corning). hADSCs were cultured in extract, and their proliferation was assessed on Days 1, 3, 5, 7 with Cell Counting Kit (CCK)-8 (Dojindo). After incubating at 37°C for 2.5 h, the absorbance of samples was measured at a wavelength of 450 nm using an MP reader (Thermo).

### Quantitative real-time polymerase chain reaction

Gene expression related to angiogenic growth factors was evaluated by qRT-PCR. hADSCs (1 × 10^5^) were seeded into PADM, FADM, LADM and FLADM of a well size of a 24-well plate and cultured for 48 h. Total RNA was extracted and reverse transcribed into cDNA with PrimeScript RT Master Mix using RNA purification kit and Reverse Transcription kit (with DNase) (EZBioscience). Gene expression levels of vascular endothelial growth factor (VEGF), insulin-like growth factor 1 (IGF-1) and epidermal growth factor (EGF) [24] were normalized to that of GAPDH and quantified with the comparative Ct method. The primers synthesized by Sangon Biotech Co. (Shanghai, China) are mentioned in Table 2.

### *In vitro* experiments of paracrine products extracted from four materials

hADSCs (1 × 10^5^) were seeded on PADM, FADM, LADM and FLADM and cultured in 1-ml culture medium. To collect the conditional medium (CM), the cells were first cultured in complete DMEM for 24 h, washed three times with PBS, then cultured in serum-free DMEM for 24 h. The supernatant was collected and centrifuged at 3000 rpm for 8 min to remove dead cells and debris.

#### Enzyme-linked immunosorbent assay

The concentrations of VEGF in CM from four materials were assessed by an enzyme-linked immunosorbent assay kit ((Multi Sciences, China) according to the manufacturer’s recommendations. The absorbance was quantified at 450 nm.

#### HUVECs proliferation and tube formation assay

The proliferation of HUVECs cultured in 50% CM and 50% serum-free endothelial culture medium was measured by Cell Counting Kit (CCK)-8 (Dojindo) after 1, 3 days of culture. For the tube formation assay, HUVECs (1 × 10^4^/well) were seeded into Matrigel-coated (Corning) 96-well plate and treated with 50% CM and 50% serum-free endothelial culture medium, and then examined by bright-field microscopy at 4h.

### *In vitro* preculture of PADM, FADM, LADM and FLADM with hADSCs before implantation

hADSCs (1 × 10^5^) resuspended in 100-µl culture medium were seeded on disinfected PADM, FADM, LADM and FLADM (in size of a well of the 24-well plate) and cultured for 4 h before adding another 1-ml DMEM supplemented with 10% FBS. The hADSCs-material compound was co-cultured for 1 week before being transplanted into nude mice.

### Implantation surgery

All procedures were approved by the Ethics Committee of Shanghai Ninth People’s Hospital (assurance no. SH9H-2021-A091-SB). Thirty-two 6-week-old male nude mice were randomly divided into eight groups (four materials, and two timepoints, *n* = 4, with two animals used for mechanical testing). After 1 week of acclimatization to the laboratory conditions, the mice were anesthetized by inhalation of isoflurane (oxygen flowmeter of 400 ml/min), and their dorsal surface were disinfected. In the experiment group, a 1.5 × 1.5 cm circular-shaped skin flap on the back of mice was lifted and the pretreated-material construct was implanted and fixed on the subcutaneous fascia. The incision was closed, and mice were allowed to recover from anesthesia.

### Macroscopic, mechanical evaluation and histological analysis

Animals were anesthetized postoperatively at 2 and 4 weeks, and the gross features were examined and recorded with a single-lens reflex camera (Nikon, Japan). A horizontal incision was made below the implanted material, the local skin flap was lifted. Then the materials and surrounding skin or muscle were harvested and fixed in 4% paraformaldehyde overnight. The samples were cut into sections and embedded in paraffin blocks.

The samples were stained with H&E stain (Solarbio, China) according to the manufacturer’s recommendation. For the evaluation of cell infiltration, three random regions of H&E staining from the upper, middle and lower parts of one slice were selected and counted. In addition, a region in size of 200 μm × 200 μm was regulated, and one region should be selected within the laser-made hole. The average number of the three regions was regarded as the final result of the sample. For better display of the implanted material, the result of Masson’s trichrome (Solarbio) was supplemented.

For immunohistochemical staining, the sections were incubated with anti-CD31 (Abcam, USA) at 1:500 dilution. The bound antibodies were 3,3′-diaminobenzidine (Beyotime Biotechnology), and the slices were counterstained with hematoxylin. The number of new blood vessels was calculated based on three random fields at 400× magnification selected from each sample.

Mechanical properties of the samples (*n* = 3) with 15 mm in length and 8 mm in width were measured using Instron Mechanics Analyzer (Model 5542, Instron, USA). The samples were stretched at a rate of 10 mm/min until fracture [25]. The analyzer recorded real-time strain and stress, and Young’s modulus and the maximum load were further calculated from the stress–strain curve using Origin software (OriginLab, USA).

### Statistical analysis

All quantitative results are presented as the mean ± standard error of mean. Statistical comparisons were performed using Student’s *t*-test, one-way analysis of variance or two-way analysis of variance followed by Tukey’s post hoc test of data or Bonferroni’s method from three independent experiments. GraphPad Prism version 8.0 software (GraphPad, Inc., La Jolla, CA, USA) was used to analyze the data. Statistical significance was set at *P* < 0.05.

## Results and discussion

### Characterization of FADM

**Figure 2.**
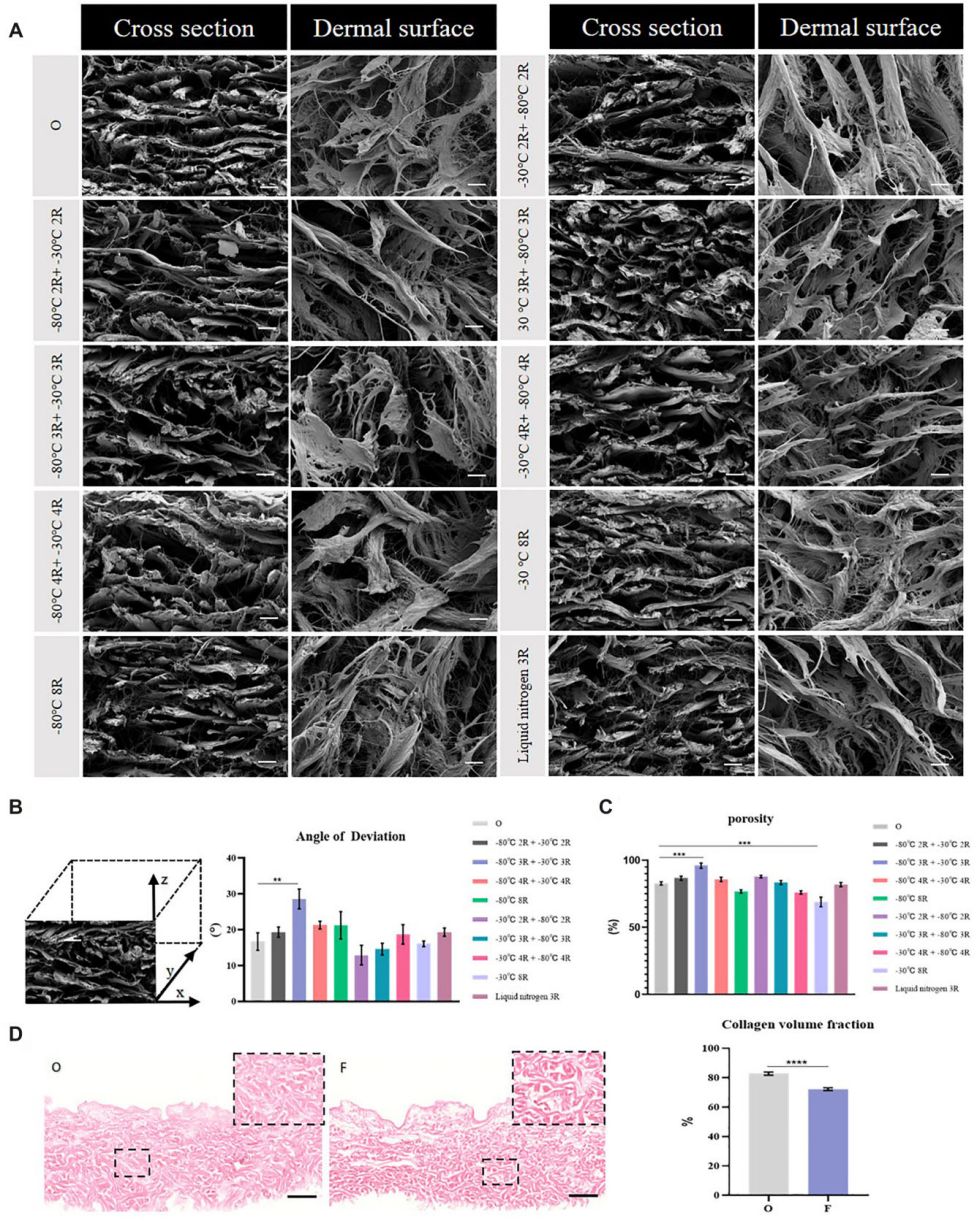
Characterization of freezing ADM. (**A**) SEM images of PADM(O) and different FADM, bar = 20 μm. (**B**) Schematic diagram of the angle of deviation (lift) and analysis of the angle of deviation (right) (*n* = 3 for each group). (**C**) Porosity and its comparison of different treatments (*n* = 3 for each group). (**D**) HE staining results for PADM (O) and FADM (F) (left) and calculation of their collagen volume fraction (right) (*n* = 3 for each group), bar = 200 μm. *P < 0.05, **P < 0.01, ***P < 0.001, ****P < 0.0001.

Inspired by frozen bean curd, a manufacturing process using freeze–thaw approach for a porous ADM was proposed that allows for easier cell infiltration and angiogenesis. In general, the solution starts to form ice crystals in conditions of −15°C to −20°C. Based on previous studies and to retain the original bio-properties, we selected freezing temperature of −30°C, −80°C or −196°C (liquid nitrogen) and a water bath of 37°C for thawing. Ten experimental groups with different temperature gradients and cycles were then established (Table 1). Since ADM is a two-sided structure with a base membrane surface and a dermal surface, and the dermal surface is prone to vessel growth, the microstructure of the cross-section and the dermal surface of FADM were observed (Fig. 2A). In PADM (Group O), the collagen fibers were arranged closely and arrayed in a parallel manner along the cross-section, and the pores were small and scattered on the dermal surface. In contrast, fibers in the FADM (Group F) were disorderly arranged, without parallel arrangement between layers, the number and diameter of pores on the dermal surface increased, and the orientation of fibers changed from unidirectional to multidirectional.

The angle of deviation was used to quantitatively analyze the distribution order of collagen fibers along the cross-section. We designated the layer parallel to a horizontal arrangement as 0°, and the layer perpendicular to the horizontal arrangement as 90°. The higher level of disorder arrangement, the closer the angle of deviation is to 45°. The average angle of deviation of Group O was 16.7°, and the average angle of deviation of ADM treated by −80°C 3R+ −30°C 3R was largest (28.6°), which yielded a significant difference compared with Group O (Fig. 2B). Thus, the layer arrangement of ADM treated by −80°C 3R + −30°C 3R was most disorderly.

The method of ethanol adsorption was used to measure the porosity of ADM treated by different freeze–thaw treatments with the same volume. Greater porosity of ADM correlated with more internal pores and looser structure. With the exception for the Group of −30°C 8R, the porosity of all other groups was higher than 70%, among which the group of −80°C 3R + −30°C 3R had the greatest porosity (96%), a significant difference compared to group O. Based on above data, we concluded that the group of −80°C 3R + −30°C 3R presented the most optimal treatment among the 10 groups for most porous FADM. FADM in subsequent experiments thus refers to ADM treated with −80°C 3R + −30°C 3R. In addition, HE staining was performed on PADM and FADM (longitudinal section), and both remained homogenous red indicating freeze–thaw treatments preserved the bio-properties of ADM. Quantitative analysis of collagen volume fraction per unit area at 200× high magnification also showed that collagen volume fraction of PADM was higher than FADM, which verified the effectiveness of freeze–thaw treatment.

Our study found that both cycles of freeze–thaw and temperature gradient influenced the connectivity of pores and the total porosity of ADM. Greater temperature gradient correlated to the faster ice crystal formation, more uniform crystal distribution, and smaller crystal nuclei. The maximum porosity from −80°C 3R + −30°C 3R treatment may be seen as ‘small crystals paved the road, and large crystals expanded the road’. The small ice crystals formed at a greater temperature gradient distributed evenly and enlarged the pores in multiple directions, while large crystals formed at a smaller temperature gradient finished the connection between pores. Our results also suggested that there was no direct interaction between cycles of freeze–thaw and looseness of biomaterials.

### Adhesion, proliferation and angiogenesis of HUVECs on PADM and FADM *in vitro*

**Figure 3.**
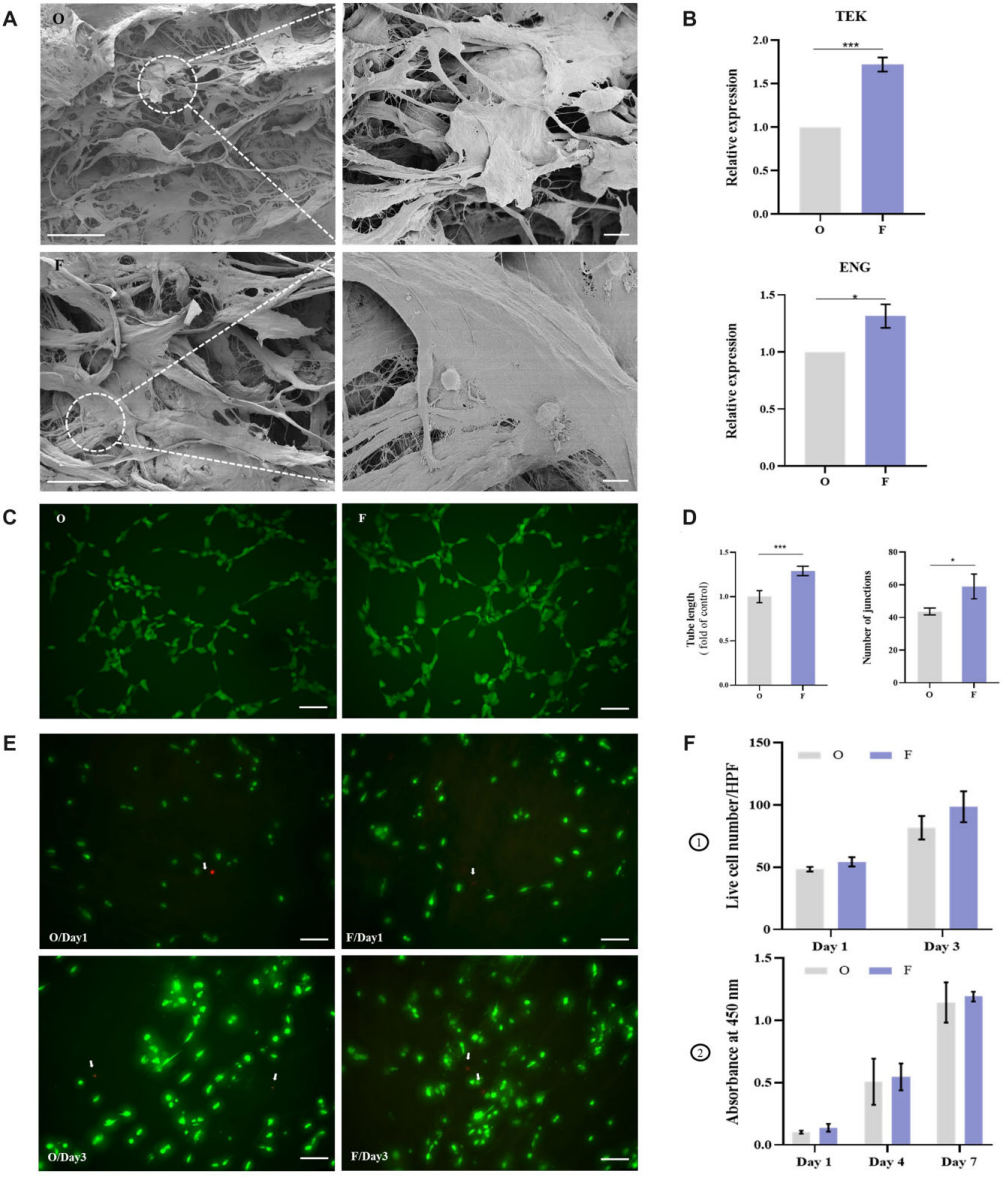
HUVECs on PADM and FADM. (**A**) SEM Images of HUVECs on PADM (O) and FADM (F), bar in the left column = 100 μm and bar in the right column = 10 μm. (**B**) The relative expression levels of Tie-2 (TEK) and CD105 (ENG) between HUVECs on PADM and FADM (*n* = 3 for each group). (**C**) Representative images of HUVECs forming tubes on PADM (O) and FADM (F) after 6 h, bar = 100 μm. (**D**) Total tube length and number of junctions of HUVECs on PADM (O) and FADM (F) (*n* = 3 for each group). (**E**) Live and dead staining of HUVECs on PADM (O) and FADM (F), white arrows represent dead cells, bar = 100 μm. (**F-1**) Calculation of live HUVECs on PADM (O) and FADM (F) for 1, 3 days. (**F-2**) Proliferation of HUVECs cultured on PADM (O) and FADM (F) for 1, 4 and 7 days (*n* = 3 for each group). *P < 0.05, **P < 0.01, ***P < 0.001, ****P < 0.0001.

The study of adhesion, proliferation and tube formation of HUVECs on PADM and FADM is helpful to evaluate the effects of freeze–thaw treatment on angiogenesis [26]. SEM showed that HUVECs adhered to PADM (group O) and FADM (group F) and extended pseudopodia. The adhesion morphology of HUVECs on the bundle and plane displayed spindle shape and sphere shape respectively (Fig. 3A). RT-PCR was used to quantitatively analyze the gene expression levels of proliferation (CD105/ENG) and function performance (Tie-2/TEK) of HUVECs. Angiopoietin-Tie2 signaling plays a critical role in morphogenesis and homeostasis of blood vessels and in vascular remodeling [27]. CD105 is predominantly expressed on proliferating HUVECs [28]. Compared with group O, the gene expressions of Tie-2 and CD105 were up-regulated in group F (Fig. 3B). Gene expressions of Tie-2 and CD105 in group F were 1.72 times and 1.31 times higher than those in group O, respectively, indicating that the proliferation and function performance of HUVECs on FADM were enhanced. Tube formation experiments were performed on PADM and FADM coated with matrix glue for 6 h (Fig. 3C). The total tube length and number of junctions of group F were significantly greater than those in group O (Fig. 3D), indicating that FADM has a stronger ability to form tubes than PADM. The proliferation of HUVECs on PADM and FADM was analyzed by live/dead staining and CCK8 assay. The survival and death of cells on PADM and FADM on Days 1 and 3 were shown in Fig. 3E. More HUVECs adhered on the surface with an even distribution in group O and group F on Day 3 than those on Day 1. The number of live cells per high power (100×) was calculated. HUVECs in group F were more than those in group O on days 1 and 3 (Fig. 3F-1). The OD values of group O and group F on Days 1, 4 and 7 were measured under 450 nm (Fig. 3F-2). Within 7 days, the OD values of the two groups continuously increased, and OD values of group F were consistently higher than group O. Therefore, HUVECs on FADM presented a trend of faster proliferation and more adhesion.

Endothelial cells (ECs) exhibited differences in adhesion, proliferation and tube formation on varied dermal templates, and vascularization of dermal templates was the key factor in successful engraftment and integration [29]. Thus, promoting ECs adhesion, proliferation and tube formation on ADM is beneficial to achieving desired therapeutic results. We simulated the HUVECs behaviors on PADM and FADM *in vitro*. It was found that gene expressions of Tie-2 and CD 105 of HUVECs on FADM were higher. HUVECs on FADM also had a stronger ability for proliferation and tube formation, which was consistent with increased gene expressions. All in all, freeze–thaw treatment promotes vascularization of ADM. We hypothesized that the enhancement of HUVECs function on FADM was related to the increase in total porosity. It has been found that HUVECs migrated to the inside of the granular material in a void fraction-dependent manner [15]. Moreover, rat smooth muscle cells could proliferate and infiltrate the scaffold with high porosity easily [30]. With a higher total porosity of FADM, more space for HUVECs adherence was present, allowing for nutrients and metabolic wastes to be easily inputted and discharged, thus enhancing cell vitality.

### Biocompatibility of treated-ADM and stemness identification of hADSCs

ADSCs served as usual tools for vascularization in tissue engineering because of their convenient management, ability to self-renew and differentiate into various cell types, and secretion of angiogenic factors [31, 32]. ADSCs-seeded silk fibroin scaffolds or hydrogels that accelerated angiogenesis were reported [33, 34]. Our previous study also reported that ADSCs-seeded-PADM promoted pro-vascular gene expression and increased vascular infiltration [35]. Thus, hADSCs were seeded on treated-ADM to strengthen vascularization. In addition, we previously reconstructed ADM by laser-micropore technique and found that LADM improved cell infiltration. Based on our previous work, we set up four groups in this study, including PADM-hADSCs, FADM-hADSCs, LADM-hADSCs and FLADM-hADSCs, to explore the effect of triple treatments combining freeze–thaw, laser drilling and hADSCs pre-culture on vascularization and cell infiltration.

**Figure 4.**
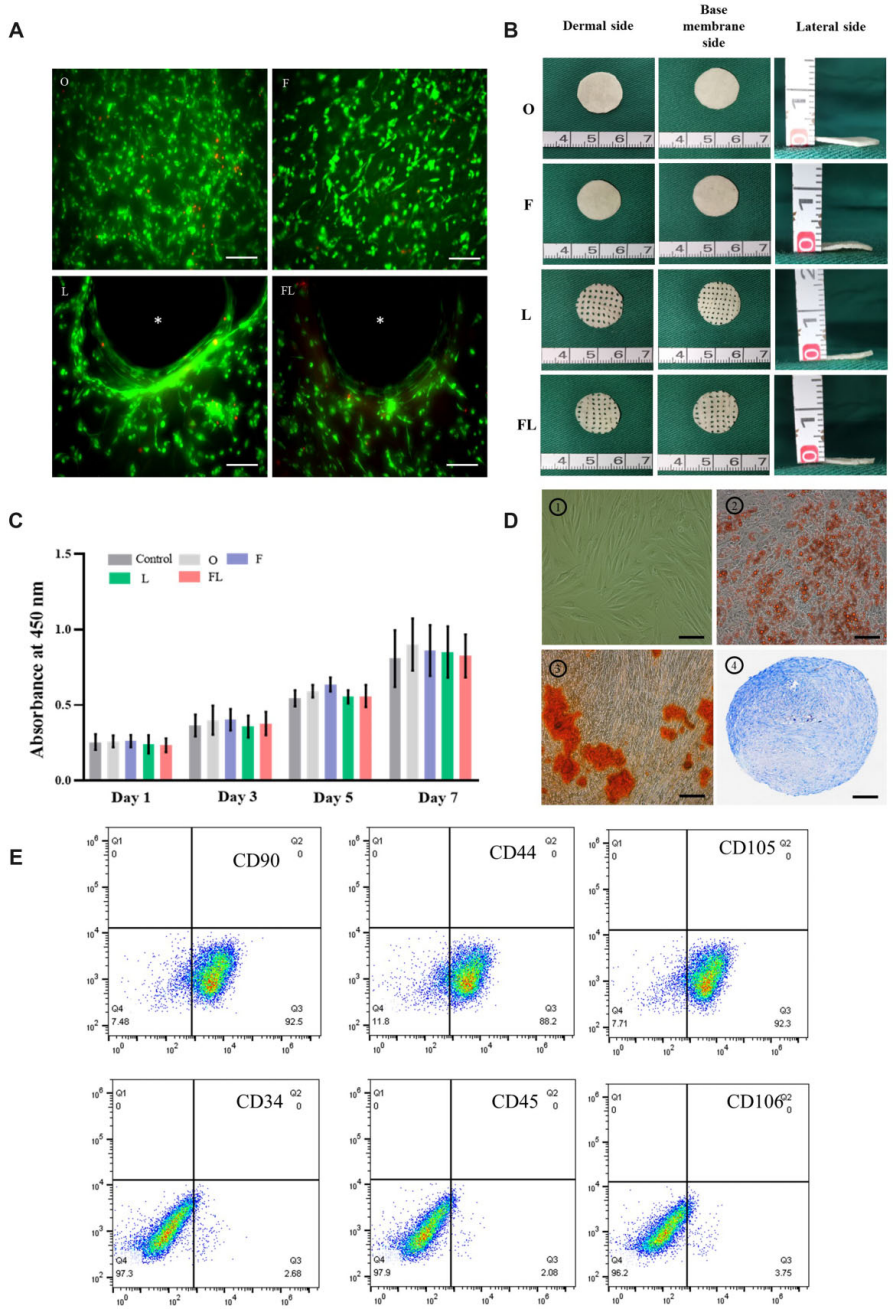
Biocompatibility of treated-ADM and stemness identification of hADSCs. (**A**) hADSCs adhesion on PADM (O), FADM (F), LADM (L) and FLADM (FL) after 2-day co-culture, * presents the pore produced by laser micropore technique, bar = 100 μm. (**B**) Gross views of PADM (O), FADM (F), LADM (L) and FLADM (FL) showing the dermal side (left), base membrane side (Middle) and lateral side (right). (**C**) hADSCs were cultured in the extracts from PADM (O), FADM (F), LADM (L) and FLADM (FL) or in complete DMEM, and the proliferation was analyzed on 1, 3, 5 and 7 days (*n* = 3 for each group). (**D**) Morphology and differentiation potential of hADSCs, bar = 200 μm. (**E**) Flow cytometry analysis.

The pores in LADM (group L) and FLADM (group FL) were introduced by advanced laser drilling technology without heat damage to surrounding tissues around the pores (Supplementary Fig. S3A). Under the acuate parameter, Laser drilling only changed the gross structure by mechanical force without microstructure changes. The adhesion of hADSCs on PADM (group O), FADM (group F), LADM (group L) and FLADM (group FL) after 2 days proliferation was shown by Live/Dead staining. hADSCs were evenly distributed on the surface of PADM and FADM with scattered cellular clusters and few dead cells. hADSCs were distributed around the pore edge on LADM and FLADM, and positions closer to the pore correlated to a denser distribution (Fig. 4A). The adhering morphology of hADSCs was further observed using SEM and TRITC-tagged-phalloidin staining (Supplementary Fig. S3B and C). Those adhering hADSCs exhibited spindle-like shape, elongated and extended to poles. The dermal side, basal membrane side and lateral side of PADM, FADM, LADM and FLADM were shown in Fig. 4B. The base membrane surface was smooth with lines, while the dermis side was rough. Round holes in a regular arrangement were observed after applying the laser micropore technique. The four materials were lyophilized and disinfected with ethylene oxide. The cytotoxicity of 72 h-extract solution from four materials was determined by cck8 assay. hADSCs cultured in low glucose complete medium were used as control, and the OD values of the five groups had no significant difference on 1, 3, 5 and 7 days (Fig. 4C). Therefore, the extract solution from PADM, FADM, LADM and FLADM did not decrease the proliferation and viability of hADSCs, which indirectly proved that the treated ADM sterilized by ethylene oxide had no cytotoxicity. Stemness identification of hADSCs was performed. The hADSCs (P3) showed a typical spindle shape under the light microscope. hADSCs could be induced to adipocytes (lipid droplets stained red), osteoblasts (calcium deposits stained red) and chondrocytes (cartilage ball stained blue) (Fig. 4D). Thus, the hADSCs have three differentiation abilities. Flow cytometry analysis [36] of the specific surface markers of hADSCs showed that CD90, CD44 and CD105 were highly expressed (92.5%, 88.2% and 92.3%, respectively), while CD34, CD45 and CD106 were negative (2.68%, 2.08% and 3.75%, respectively). CD 90, CD44 and CD105 were specific markers of stem cells. CD106 mediated immune cell adhesion and was absent or low expressed in hADSCs [37]. CD34 and CD45 are surface markers of hematopoietic cells, and low expressions of them indicated high purity of hADSCs after amplification with few hematopoietic cells.

### Gene expression and paracrine products of hADSCs-treated ADM co-culture system

**Figure 5.**
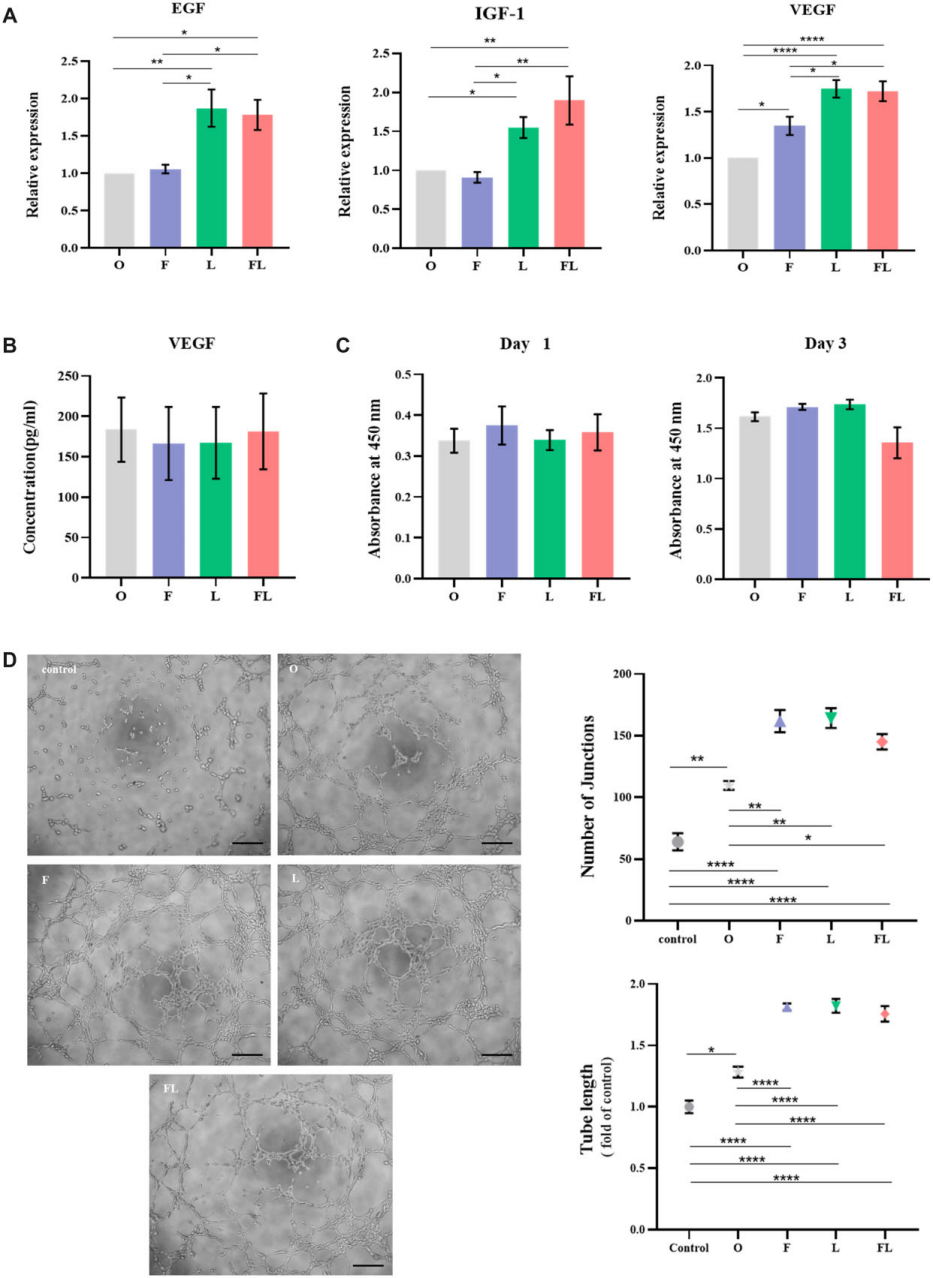
*In vitro* experiments of ADM precultured with hADSCs. (**A**) Gene expression levels of pro-angiogenic factors in O, F, L and FL groups (*n* = 3 for each group). (**B**) Levels of VEGF secreted by hADSCs in O, F, L and FL groups (*n* = 3 for each group). (**C**) Proliferation of HUVECs cultured by conditional media on 1 and 3 days (*n* = 3 for each group). (**D**) Tube formation induced by conditional media at 4 h, bar = 200 μm (left). Statistical analysis of the number of junctions and total tube length among O, F, L and FL groups (right) (*n* = 3 for each group). *P < 0.05, **P < 0.01, ***P < 0.001, ****P < 0.0001.

hADSCs were seeded on the PADM (group O), FADM (group F), LADM (group L) and FLADM (group FL), and co-cultured for 2 days. RNA of hADSCs was extracted and the gene expressions of pro-angiogenesis (EGF, IGF-1, VEGF) were quantitatively analyzed. The gene expressions of group L and group FL were significantly higher than those in group O and F. The expressions of VEGF in group F, L and FL were significantly higher than that in group O. The difference in gene expressions between groups L and FL were not statistically significant. The expressions of EGF, IGF-1 and VEGF in group FL were 1.78, 1.90, and 1.72 times higher than those in group O, respectively. Compared with group O, the other three groups increased the pro-angiogenesis gene of hADSCs expression (Fig. 5A). However, there was no statistical difference in VEGF, a secreted protein in the supernatant from four co-culture systems (Fig. 5B). It may be caused by the complex mechanism of gene expression and protein translation of VEGF. Part of VEGF proteins reminds bound to the extracellular matrix resulting in inconsistent outcomes with the PCR results [38].

The effects on proliferation and tube formation of HUVECs of 4 co-culture systems were further invested. HUVECs were cultured in four CMs (50% supernatant from co-culture system and 50% serum-free endothelial medium), and there were no significant differences in proliferation among the four groups on 1 and 3 days (Fig. 5C). In terms to tube formation, HUVECs were cultured in serum-free endothelial medium (control group) and four CMs (Fig. 5D). After 4 h, there were no obvious ring-shaped tubes and merely few scattered junctions in the control group. Group O, F, L and FL all displayed more tubes and more junctions with uniform distribution. Either the number of junctions or the total tube length, the control group was the lowest with a statistical difference. Furthermore, the junctions and tube length in group F, L and FL were also significantly larger than those on group O, but no statistical difference was found among the above three groups.

Physiological angiogenesis is a complicated process that depends on the vascular microenvironment regulated by positive and negative angiogenic modulators and requires functional activities of other molecules like extracellular matrix proteins and adhesion receptors [39]. The cell-scaffold co-culture system could provide a pro-angiogenesis microenvironment by paracrine function [35, 40]. In addition, hADSCs behaved differently in materials with various porosity. hADSCs performed higher adhesion, proliferation and cell interaction within high porosity nanofibrous scaffolds that provided a well microenvironments [41]. Higher cell viability and spreading morphology of hADSCs were observed in the aerogel with more porosity as well [42]. Our previous studies also found that laser perforation does not alter the characteristics of PADM [14], and the expression and secretion of pro-angiogenic factors by ADSCs are significantly increased. Thus, we further investigated the pro-angiogenesis microenvironment produced by hADSCs on FLADM. VEGF, IGF-1 and EGF are pro-angiogenic factors and play an important role in angiogenesis. VEGF promotes the growth of vascular ECs, angiogenesis and is also a survival factor for ECs [43]. IGF-1 is a cell surface receptor that regulates cell growth and metabolism [44]. EGF is also a regulator of angiogenesis [45]. Compared with hADSCs on PADM, the hADSCs on FADM, LADM and FLADM highly expressed EGF, IGF-1 and VEGF. The proliferation of HUVECs yielded no statistical difference among the four co-culture systems. However, there were differences in promoting tube formation on four co-culture systems, among which PADM-hADSCs co-culture system had the worst performance, and the other three systems demonstrated no significant difference. In conclusion, the porosity of the AMD has a quiet influence on pro-angiogenic microenvironment formed by its co-culture system due to cell behavior change. The FLADM-hADSCs co-culture system yielded no obvious advantage in promoting proliferation of HUVECs, but showed a stronger ability to form tubes compared with the PADM-hADSCs system. All in all, FLADM-hADSCs co-culture system provided a pro-vascular microenvironment.

### *In vivo* experiment and the changes in mechanical properties

**Figure 6.**
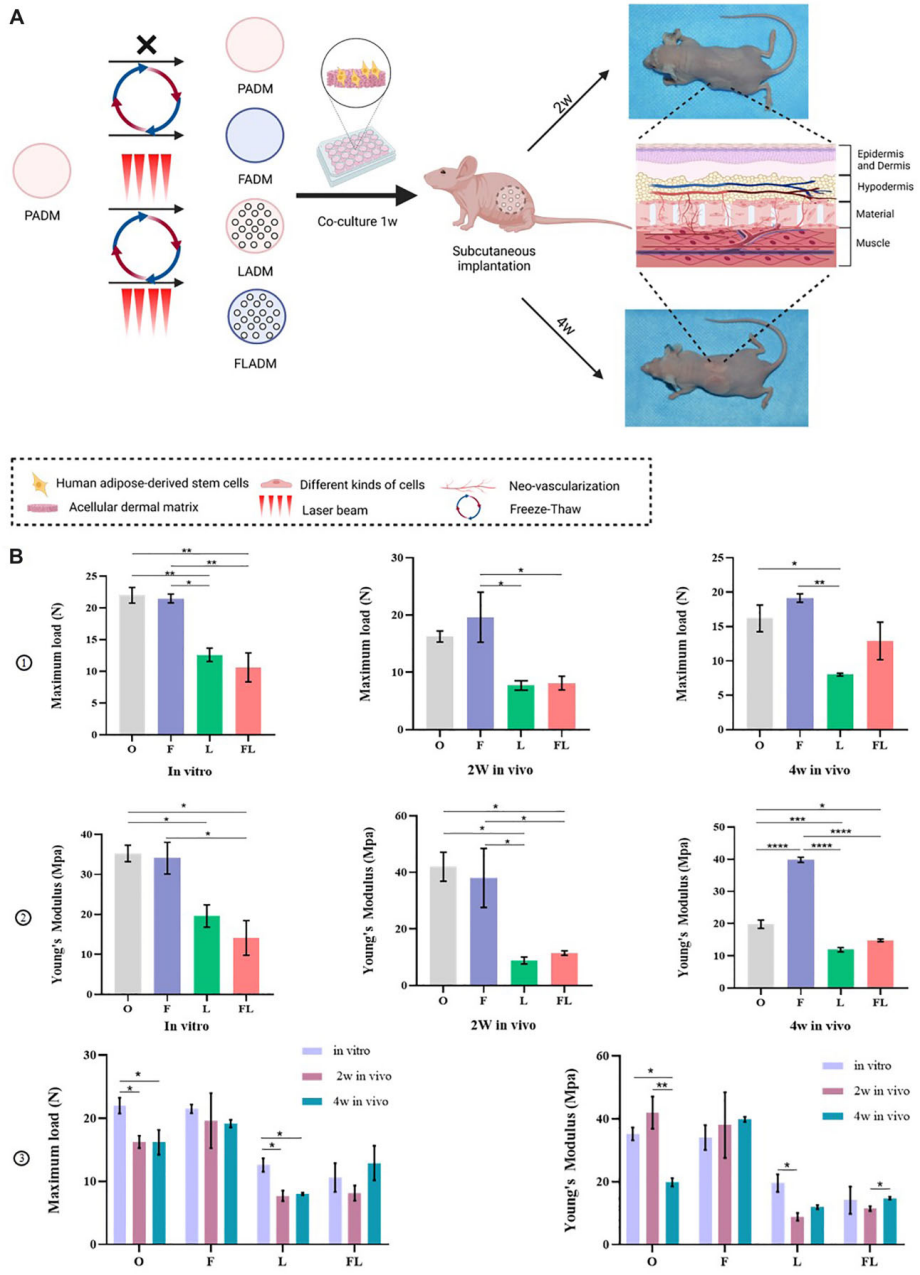
*In vivo* experiment and mechanical properties. (**A**) The schematic diagram of animal experiments created by BioRender.com. （**B**） The maximum load and young’s modulus of PADM (O), FADM (F), LADM (L) and FLADM (FL) *in vitro*, at 2w and at 4w (*n* = 3 for each group). *P < 0.05, **P < 0.01, ***P < 0.001, ****P < 0.0001.

The schematic diagram of animal experiments is shown in Fig. 6A, which was created by BioRender.com and approved for publication. PADM, FADM, LADM and FLADM were pre-cultured with hADSCs *in vitro* for 1 week, and then embedded into the back of nude mice. Samples were taken at 2w and 4w, respectively, after operation. The detailed procedures are described in Implantation surgery section for reference. Theocratically, FLADM would have more vascular growth and cell infiltration, and blood vessels would sprout out from the punched holes or the internal pores of the material.

The biomechanics (Young’s modulus and maximum load) of PADM (group O), FADM (group F), LADM (group L) and FLADM (group FL) before and after implantation were analyzed and changes in mechanical properties were evaluated to reflect the treated-ADM remodeling process (Supplementary Fig. 6B). Figure 6B-1 showed the changes in the maximum load. Before implantation, the maximum loads of group O and F were significantly higher than those of group L and FL, while the load of group F was slightly lower than group O. At 2w, the maximum load of group F was superior to group O that was the largest among four groups. At 4w, the maximum load of group F was still the largest, and the load of group FL exceeded that of group L. Figure 6B-2 displayed the changes in Young’s modulus. Young’s modulus quantitatively describes the deformation resistance of materials. A larger modulus correlated to stronger tensile strength. Before implantation, the modulus of group O was the largest, while group FL was the smallest. At 2w, the modulus of group FL exceeded that of group L. At 4w, the modulus of group F exceeded group O achieving the highest value, and the modulus of group FL was still better than that of group L which remained at the value seen before implantation. Combined with Fig. 6B-3, in terms of the maximum load, four groups all showed a downward trend at 2w, but showed an upward trend at 4w with the largest rises in group FL. In terms of Young’s modulus, except for group O, the other three groups exhibited better performance at 4w.

The ADM underwent remodeling during various biological processes, and the mechanical properties changed accordingly [46]. The tensile test is the most widely used mechanical test performed on skin specimens [47]. As matrix remodeling progresses, the stiffness of the matrix increases [48]. The initial values of LADM and FLADM were lower than those of PADM and FADM both in the maximum load and Young’s modulus. Although the laser drilling decreased the mechanical strength, the values of Young’s modulus were all >10 Mpa, which meets the strength requirements of most tissue substitutes. Degradation of the materials and the matrix deposition to remodel materials by infiltrating cells simultaneously during the regenerative process [49, 50]. Though the stiffness and strength of a scaffold decrease with the porosity increasing, the biological processes enhanced [51]. The swelling ratios of FADM, LADM and FLADM were significantly higher than that of PADM (Supplementary Fig. S1A) due to porosity increase, indicating that the better nutrients are absorbed in the scaffolds for adhering cells [52]. In addition, the degradation rate rises with structure porosity increasing because of the enlarged surface [53]. The degradation results of the four groups were shown in Supplementary Fig. S1B. Both on 1 and 3 days, the PADM degraded most slowly, while FLADM was in the highest degradation speed. The difference in degradation rates among the four groups was mainly caused by porosity variance. Thus, the biomechanical properties decreased at first and increased subsequently, indicating that there was a balance between the material degradation and extracellular matrix deposition. The remolding process of FADM, LADM and FLADM was enhanced due to porosity increase. FADM, LADM and FLADM showed earlier mechanical properties enhancement compared with PADM, and the improvement of FLADM was the most significant. In conclusion, FLADM pre-cultured with hADSCs can achieve early remodeling initiation.

### Histological and immunohistochemical analyses

**Figure 7.**
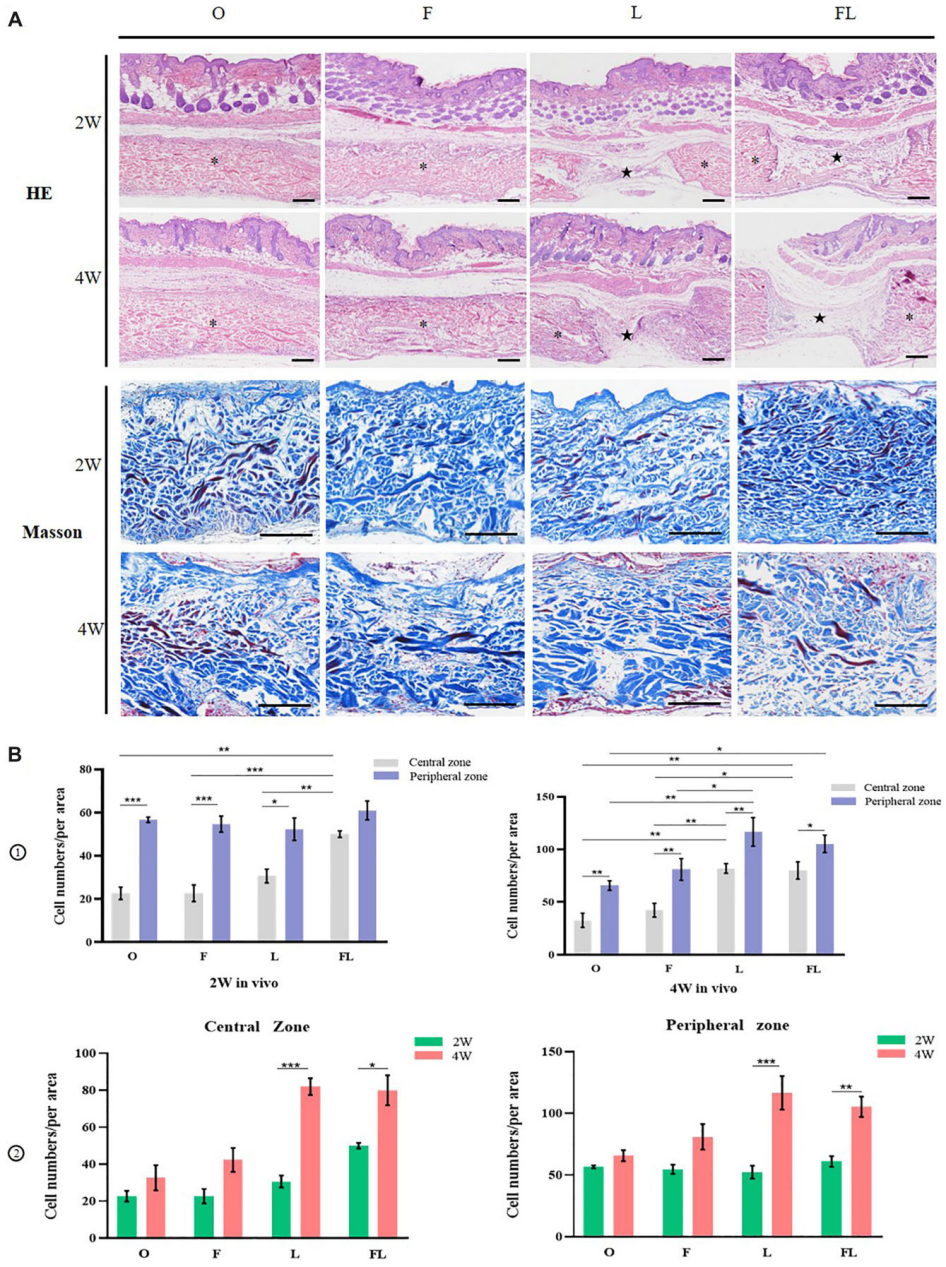
Histological staining. (**A**) HE and Masson staining of samples, bar = 200 μm (the M characters represented the implanted material, and the pentagram indicated the pore produced by laser drilling). (**B**) Statistical analysis of cell infiltration, cell numbers per area in the Central zone and peripheral zone (*n* = 3 for each group). *P < 0.05, **P < 0.01, ***P < 0.001, ****P < 0.0001.

In order to further observe the cell infiltration of PADM, FADM, LADM and FLADM (all of them pre-cultured with hADSCs), we conducted HE staining and Masson staining (Fig. 7A). HE staining highlighted the relationship between the skin and the material, while Masson staining focused on the material itself. It has been observed that cells infiltrated materials at 4w were significantly more than those at 2w. The ‘membrane-like’ autologous tissue has been seen in the perforated holes, in which cells and blood vessels increased significantly. According to the distance between material and surrounding tissue (skin layer and muscle layer), it can be divided into central zone and peripheral zone. Since the host cells generally migrated from the periphery to the central region, more cells in the central zone indicated accelerated cell migration or increased proliferation. We calculated and quantitatively analyzed the number of cells in peripheral and central zones at 2w and 4w (Fig. 7B). The size of 200 μm × 200 μm was artificially set as the counting region, three regions were randomly picked in the upper, middle and lower parts of the same slice and had their results averaged. In addition, one region should be selected in the hole for drilled samples. At 2w, there was no significant difference in the number of cells in the peripheral zone of the four groups, but the number of cells in the central zone of group FL was obviously higher than that of the other three groups. At 4w, the number of cells in the peripheral area of group L and FL was significantly higher than that of group O, and the number of cells in the central zone of group L and FL was statically higher than that of group O and F, while there was no obvious difference between group L and FL. Whether in the central or peripheral zone, the number of cells on four groups at 4w was higher than that of 2w, and groups L and FL were significantly increased.

**Figure 8.**
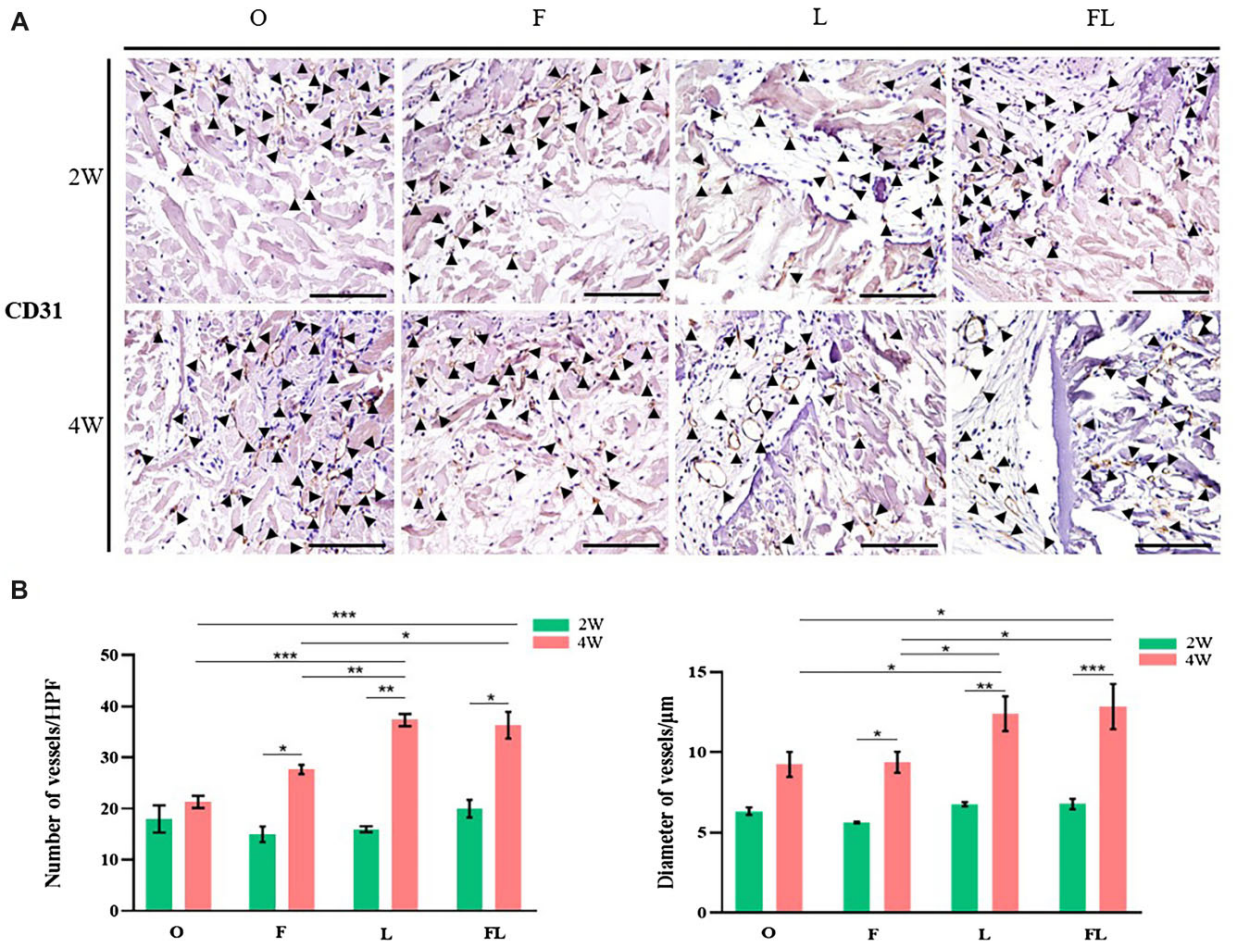
Immunohistochemical staining. (**A**) Immunohistochemical staining for CD31 in materials, black triangle indicates the new vessels, bar = 10 μm. (**B**) Number of blood vessels of per high-power field (HPF) and diameter of vessels (*n* = 3 for each group). *P < 0.05, **P < 0.01, ***P < 0.001, ****P < 0.0001.

To better demonstrate angiogenesis within materials, CD 31 on ECs was stained (brown, black triangle marked in Fig. 8A). New blood vessels were observed in the perforated pores and at the edge of the material, indicating that the closer to the autologous tissue, the greater vascular density was. Thus, increasing the contact areas with surrounding tissues is beneficial to the vascularization of the material. We counted the vessels under high magnification (400×). At 2w, there was no significant difference among the four groups. At 4w, the mean number of vessels in groups F, L and FL was higher than that in group O, and the number of vessels in groups L and FL was also significantly higher than that in group F. In addition, the number of vessels on four groups at 4w were all greater than that at 2w, with statistical differences found for groups F, L and FL. In terms of the blood vessel diameter, there was no significant difference among the four groups, which was <10 μm, belonging to capillaries (6–9 μm). At 4w, the blood vessel diameter of four groups increased. The diameters in groups L and FL were significantly larger than that found in groups O and F, which were 10–15 μm, belonging to micro veins (7–50 μm) (Fig. 8B).

Through *in vivo* experiment, we found that FLADM pre-cultured with hADSCs had the most infiltrated cells at 2w, and there were a large number of cells in the central zone. It may be directly related to the total porosity increase of ADM. Freeze–thaw treatment loosened the internal structure, providing more space for cell adhesion, and meeting the metabolic requirements of more cells. The laser drilling method significantly promoted the autologous tissue growth into ADM. Quantitative analysis of the number and diameter of new blood vessels showed that both freeze–thaw and laser drilling obviously improved the blood vessel density per area, and vessels diameter increased markedly after laser drilling, which may be related to blood vessels that sprouted from the vessels in the upper skin or lower muscle passing through holes or earlier blood vessel remodeling initiation. Thus, hADSCs seeded-FLAM increased cell infiltration and accelerated vascularization at the early stage after implantation, resulting in early remodeling initiation and better integration with surrounding tissues.

The 21st century is the era of biomaterials with immense improvements. However, the number of biomaterials applied to clinical practice is limited except for acellular tissue matrixes [54, 55]. The extracellular matrix is a unique substance produced by organisms for adapting to a special environment and surviving in evolution. Thus, the acellular tissue matrix has advantages of structural proteins with complex functions and natural 3D structures that are difficult to be simulated by synthetic materials. It has attracted extensive attention from researchers, for example, Wu *et al*. used a heparinized porcine acellular liver scaffold loaded with mesenchymal stem cells to construct liver tissue [56]. The ADM was one of the acellular tissue matrices with high clinical value as tissue filler or soft tissue substitute [32]. However, it was found that ADM was difficult to achieve good reconstruction results in patients with diabetic foot ulcers due to local ischemic and insufficient blood supply to ADM from long-term clinical follow-up. In addition, ADM resurfaced by skin graft is not always possible and indicated, such as for extensive burns. Thus, in order to reduce the incidence of early complications related to ADM usages, like seroma and implant necrosis, and to relieve foreign body sensation caused by insufficient cell infiltration and poor integration with surrounding tissue, it is urgent to promote early cell infiltration and vascularization of ADM. In this study, we constructed ADM by a triple treatment combining freeze–thaw, laser drilling and hADSCs pre-culture. In vitro, we found that gene expression of pro-angiogenic factors increased, and the ability to form tubes strengthened. In vivo experiment further verified the feasibility of the measure. We observed greater cell infiltration, especially on the central region, more blood vessels and bigger vessel diameter. Therefore, the triple treatment promotes cell adhesion, migration, proliferation and new blood vessel growth. All in all, the advantages of this strategy can be summarized as follows.

- Fast, convenient and easy to control. Nowadays, laser micropore technique has become more mature [57], and freeze–thaw conditions are easy to achieve and control. In addition, the factory production of cell large-scale cultivation has been realized. Therefore, the preparation process is short in time and viable for mass production.
- Maintaining original micro-structure and satisfying mechanical requirements. Compared with chemical crosslinking, the physical modification does not destroy the natural components of ADM with higher biological safety, and retains its micro-3D structure for cell adhesion, proliferation, differentiation and maturation. It was found that the biomechanical properties of ADM decreased a little at the early incorporation stage, but with development of adaptive remodeling, the properties gradually recovered to the initial level or even exceeded the original value, meeting the mechanical requirements of the replaced tissue.
- Increasing the total porosity of ADM, improving its vascularization and cellular carrier capacity, further enhancing its clinical application potential. Freeze–thaw treatment significantly improved the density of internal structure with total porosity increasing, providing more areas for cell adhesion and proliferation. hADSCs on FLADM highly expressed genes of pro-angiogenic factors and provide a better microenvironment for tube formation. More cell infiltration and blood vessels were also observed *in vivo*. Thus, the triple pretreatment promotes early vascularization and integration with host tissues, which is conducive to improving therapeutic results and the postoperative satisfaction of patients.

## Conclusion

Freeze–thaw treatment is one of the more effective measures to optimize the internal structure of ADM, and also benefits adhesion, proliferation and tube formation of HUVECs. In the present study, the porcine dermal matrix fabricated by laser micropore technique and freeze–thaw cycles (−80°C 3R + −30°C 3R) were co-cultured with hADSCs to achieve early integration. This novel ADM with tri-treatments could not only enhance the gene expression levels of pro-angiogenic factors and tube formation capacity, but also promote cell infiltration and angiogenesis. In conclusion, with the advantages of fast, convenient and easy to control manufacturing, along with the preservation of micro-structure and mechanical properties, and promotion of early vascularization and integration, the FLADM-hADSCs co-cultured system might inspire further clinical adoption of heterologous ADM.

## Supplementary data

Supplementary data are available at *REGBIO* online.

## Supplementary Material

rbac053_Supplementary_Data

## References

[1] Zhang Y , Chen Y, Zhao B, Gao J, Xia L, Xing F, Kong Y, Li Y, Zhang G. Detection of type I and III collagen in porcine acellular matrix using HPLC-MS. Regen Biomater 2020;7:577–82. 33365143 10.1093/rb/rbaa032 PMC7748446

[2] Da LC , Huang YZ, Xie HQ, Zheng BH, Huang YC, Du SR. Membranous extracellular matrix-based scaffolds for skin wound healing. Pharmaceutics 2021;13:1796. 34834211 10.3390/pharmaceutics13111796 PMC8620109

[3] Patel S , Ziai K, Lighthall JG, Walen SG. Biologics and acellular dermal matrices in head and neck reconstruction: a comprehensive review. Am J Otolaryngol 2022;43:103233. 34537508 10.1016/j.amjoto.2021.103233

[4] Łabuś W , Kitala D, Szapski M, Klama-Baryła A, Kraut M, Smętek W. Tissue engineering in skin substitute. Adv Exp Med Biol 2021;1345:193–208. 34582024 10.1007/978-3-030-82735-9_16

[5] Tork S , Jefferson RC, Janis JE. Acellular dermal matrices: applications in plastic surgery. Semin Plast Surg 2019;33:173–84. 31384233 10.1055/s-0039-1693019 PMC6680075

[6] Kim J , Song SY, Lee SG, Choi S, Lee YI, Choi JY, Lee JH. Treatment of human immunodeficiency virus-associated facial lipoatrophy with hyaluronic acid filler mixed with micronized cross-linked acellular dermal matrix. J Korean Med Sci 2022;37:e37. 35132843 10.3346/jkms.2022.37.e37 PMC8822113

[7] Ohlinger R , Nawroth F, Kohlmann T, Alwafai Z, Schueler K, Zygmunt M, Paepke S. Retrospective study of radiotherapy impact on the outcome of material-assisted implant-based subpectoral breast reconstruction. Anticancer Res 2021;41:2017–24. 33813408 10.21873/anticanres.14969

[8] Russello D , Sofia M, Conti P, Latteri S, Pesce A, Scaravilli F, Vasta F, Trombatore G, Randazzo V, Schembari E, Barchitta M, Agodi A, La Greca G. A retrospective, Italian multicenter study of complex abdominal wall defect repair with a Permacol biological mesh. Sci Rep 2020;10:3367. 32099052 10.1038/s41598-020-60019-0 PMC7042221

[9] Romain B , Story F, Meyer N, Delhorme JB, Brigand C, Rohr S. Comparative study between biologic porcine dermal meshes: risk factors of postoperative morbidity and recurrence. J Wound Care 2016;25:320–5. 27286664 10.12968/jowc.2016.25.6.320

[10] Patel KM , Bhanot P. Complications of acellular dermal matrices in abdominal wall reconstruction. Plast Reconstr Surg 2012;130:216s–24s. 23096976 10.1097/PRS.0b013e318262e186

[11] Komatsu I , Yang J, Zhang Y, Levin LS, Erdmann D, Klitzman B, Hollenbeck ST. Interstitial engraftment of adipose-derived stem cells into an acellular dermal matrix results in improved inward angiogenesis and tissue incorporation. J Biomed Mater Res 2013;101:2939–47. 10.1002/jbm.a.34582 23554077

[12] Iyyanki TS , Dunne LW, Zhang Q, Hubenak J, Turza KC, Butler CE. Adipose-derived stem-cell-seeded non-cross-linked porcine acellular dermal matrix increases cellular infiltration, vascular infiltration, and mechanical strength of ventral hernia repairs. Tissue Eng Part A 2015;21:475–85. 25156009 10.1089/ten.tea.2014.0235 PMC4334099

[13] Lee J , Lee S, Ahmad T, Madhurakkat Perikamana SK, Lee J, Kim EM, Shin H. Human adipose-derived stem cell spheroids incorporating platelet-derived growth factor (PDGF) and bio-minerals for vascularized bone tissue engineering. Biomaterials 2020;255:120192. 32559565 10.1016/j.biomaterials.2020.120192

[14] Zhu Z , Yuan ZQ, Huang C, Jin R, Sun D, Yang J, Luo XS. Construction of a dermis-fat composite in vivo: optimizing heterogeneous acellular dermal matrix with in vitro pretreatment. J Tissue Eng Regen Med 2020;14:215–28. 31729841 10.1002/term.2986

[15] Seymour AJ , Shin S, Heilshorn SC. 3D printing of microgel scaffolds with tunable void fraction to promote cell infiltration. Adv Healthc Mater 2021;10:e2100644. 34342179 10.1002/adhm.202100644 PMC8612872

[16] Pach E , Verdaguer A. Studying ice with environmental scanning electron microscopy. Molecules 2021;27:258. 35011490 10.3390/molecules27010258 PMC8746807

[17] Zhang, H. Introduction to Freeze-drying and Ice Templating. In: H. Zhang (Ed.). *Ice Templating and Freeze‐Drying for Porous Materials and Their Applications* 2018;1–27.

[18] Cheng Y , Lu S, Hu Z, Zhang B, Li S, Hong P. Marine collagen peptide grafted carboxymethyl chitosan: optimization preparation and coagulation evaluation. Int J Biol Macromol 2020;164:3953–64. 32898540 10.1016/j.ijbiomac.2020.09.006

[19] Wahab AHA , Saad APM, Harun MN, Syahrom A, Ramlee MH, Sulong MA, Kadir MRA. Developing functionally graded PVA hydrogel using simple freeze-thaw method for artificial glenoid labrum. J Mech Behav Biomed Mater 2019;91:406–15. 30684888 10.1016/j.jmbbm.2018.12.033

[20] Khorasani MT , Joorabloo A, Adeli H, Mansoori-Moghadam Z, Moghaddam A. Design and optimization of process parameters of polyvinyl (alcohol)/chitosan/nano zinc oxide hydrogels as wound healing materials. Carbohydr Polym 2019;207:542–54. 30600038 10.1016/j.carbpol.2018.12.021

[21] Canadas RF , Costa JB, Mao Z, Gao C, Demirci U, Reis RL, Marques AP, Oliveira JM. 3DICE coding matrix multidirectional macro-architecture modulates cell organization, shape, and co-cultures endothelization network. Biomaterials 2021;277:121112. 34488122 10.1016/j.biomaterials.2021.121112

[22] Guo P , Du P, Zhao P, Chen X, Liu C, Du Y, Li J, Tang X, Yang F, Lv G. Regulating the mechanics of silk fibroin scaffolds promotes wound vascularization. Biochem Biophys Res Commun 2021;574:78–84. 34438350 10.1016/j.bbrc.2021.08.026

[23] Choi M , Sultana T, Park M, Lee BT. Fibroblast cell derived extracellular matrix containing electrospun scaffold as a hybrid biomaterial to promote in vitro endothelial cell expansion and functionalization. Mater Sci Eng C Mater Biol Appl 2021;120:111659. 33545826 10.1016/j.msec.2020.111659

[24] Qin F , Zhang W, Zhang M, Long X, Si L, Li Z, Huang J, Wang X. Adipose-derived stem cells improve the aging skin of nude mice by promoting angiogenesis and reducing local tissue water. Aesthet Surg J 2021;41:NP905–13. 33428732 10.1093/asj/sjab001

[25] Jiang S , Liu S, Feng W. PVA hydrogel properties for biomedical application. J Mech Behav Biomed Mater 2011;4:1228–33. 21783131 10.1016/j.jmbbm.2011.04.005

[26] Guo FH , Guan YN, Guo JJ, Zhang LJ, Qiu JJ, Ji Y, Chen AF, Jing Q. Single-Cell transcriptome analysis reveals embryonic endothelial heterogeneity at spatiotemporal level and multifunctions of MicroRNA-126 in mice. Arterioscler Thromb Vasc Biol 2022;42:326–42. 35021856 10.1161/ATVBAHA.121.317093 PMC8860216

[27] Jo G , Bae J, Hong HJ, Han AR, Kim DK, Hong SP, Kim JA, Lee S, Koh GY, Kim HM. Structural insights into the clustering and activation of Tie2 receptor mediated by Tie2 agonistic antibody. Nat Commun 2021;12:6287. 34725372 10.1038/s41467-021-26620-1 PMC8560823

[28] Ruiz-Llorente L , Vega MC, Fernández FJ, Langa C, Morrell NW, Upton PD, Bernabeu C. Generation of a soluble form of human endoglin fused to green fluorescent protein. *IJMS* 2021;22:11282. 34681942 10.3390/ijms222011282 PMC8539536

[29] Agostinis C , Spazzapan M, Vuerich R, Balduit A, Stocco C, Mangogna A, Ricci G, Papa G, Zacchigna S, Bulla R. Differential capability of clinically employed dermal regeneration scaffolds to support vascularization for tissue bioengineering. Biomedicines 2021;9:1458. 34680575 10.3390/biomedicines9101458 PMC8533449

[30] Do TM , Yang Y, Deng A. Porous bilayer vascular grafts fabricated from electrospinning of the recombinant human collagen (RHC) peptide-based blend. Polymers (Basel) 2021;13:4042. 34833340 10.3390/polym13224042 PMC8619216

[31] Kim YH , Im GB, Kim SW, Kim YJ, Yu T, Lee JR, Um SH, Joung YK, Bhang SH. Anti-senescence ion-delivering nanocarrier for recovering therapeutic properties of long-term-cultured human adipose-derived stem cells. J Nanobiotechnol 2021;19:352. 10.1186/s12951-021-01098-7 PMC8557526 34717632

[32] Tognetti L , Pianigiani E, Ierardi F, Lorenzini G, Casella D, Liso FG, De Pascalis A, Cinotti E, Rubegni P. The use of human acellular dermal matrices in advanced wound healing and surgical procedures: state of the art. Dermatol Ther 2021;34:e14987. 33993627 10.1111/dth.14987

[33] Watchararot T , Prasongchean W, Thongnuek P. Angiogenic property of silk fibroin scaffolds with adipose-derived stem cells on chick chorioallantoic membrane. R Soc Open Sci 2021;8:201618. 33959331 10.1098/rsos.201618 PMC8074929

[34] Kim SH , Kim D, Cha M, Kim SH, Jung Y. The regeneration of Large-Sized and vascularized adipose tissue using a tailored elastic scaffold and dECM hydrogels. Int J Mol Sci 2021;22(22):12560. 10.3390/ijms222212560 PMC8624932 34830444

[35] Zhu Z , Yuan ZQ, Huang C, Jin R, Sun D, Yang J, Luo XS. Pre-culture of adipose-derived stem cells and heterologous acellular dermal matrix: paracrine functions promote post-implantation neovascularization and attenuate inflammatory response. Biomed Mater 2019;14:035002. 30699384 10.1088/1748-605X/ab0355

[36] Yang XF , He X, He J, Zhang LH, Su XJ, Dong ZY, Xu YJ, Li Y, Li YL. High efficient isolation and systematic identification of human adipose-derived mesenchymal stem cells. J Biomed Sci 2011;18:59. 21854621 10.1186/1423-0127-18-59 PMC3175156

[37] Yang ZX , Han ZB, Ji YR, Wang YW, Liang L, Chi Y, Yang SG, Li LN, Luo WF, Li JP, Chen DD, Du WJ, Cao XC, Zhuo GS, Wang T, Han ZC. CD106 identifies a subpopulation of mesenchymal stem cells with unique immunomodulatory properties. PLoS One 2013;8:e59354. 23555021 10.1371/journal.pone.0059354 PMC3595282

[38] Ferrara N. Vascular endothelial growth factor: molecular and biological aspects. Curr Top Microbiol Immunol 1999;237:1–30. 9893343 10.1007/978-3-642-59953-8_1

[39] Ribatti D. Angiogenesis. In: Maloy S, Hughes K (eds). Brenner's Encyclopedia of Genetics *(*Second Edition). San Diego: Academic Press, 2013,130–2.

[40] Wang Q , Jin Y, Deng X, Liu H, Pang H, Shi P, Zhan Z. Second-harmonic generation microscopy for assessment of mesenchymal stem cell-seeded acellular dermal matrix in wound-healing. Biomaterials 2015;53:659–68. 25890761 10.1016/j.biomaterials.2015.03.011

[41] Khoramgah MS , Ranjbari J, Abbaszadeh HA, Tabatabaei Mirakabad FS, Hatami S, Hosseinzadeh S, Ghanbarian H. Freeze-dried multiscale porous nanofibrous three dimensional scaffolds for bone regenerations. Bioimpacts 2020;10:73–85. 32363151 10.34172/bi.2020.10 PMC7186540

[42] Xia H , Dong L, Hao M, Wei Y, Duan J, Chen X, Yu L, Li H, Sang Y, Liu H. Osteogenic property regulation of stem cells by a hydroxyapatite 3D-hybrid scaffold with cancellous bone structure. Front Chem 2021;9:798299. 34869241 10.3389/fchem.2021.798299 PMC8640089

[43] Ferrara N. Vascular endothelial growth factor: basic science and clinical progress. Endocr Rev 2004;25:581–611. 15294883 10.1210/er.2003-0027

[44] Delafontaine P. Insulin-like growth factor I and its binding proteins in the cardiovascular system. Cardiovasc Res 1995;30:825–34. 8746194

[45] Serban F , Artene SA, Georgescu AM, Purcaru SO, Tache DE, Alexandru O, Dricu A. Epidermal growth factor, latrophilin, and seven transmembrane domain-containing protein 1 marker, a novel angiogenesis marker. Onco Targets Ther 2015;8:3767–74. 26719704 10.2147/OTT.S93843 PMC4689259

[46] Alonzo M , Kumar SA, Allen S, Delgado M, Alvarez-Primo F, Suggs L, Joddar B. Hydrogel scaffolds with elasticity-mimicking embryonic substrates promote cardiac cellular network formation. Prog Biomater 2020;9:125–37. 32978746 10.1007/s40204-020-00137-0 PMC7544760

[47] Terzini M , Bignardi C, Castagnoli C, Cambieri I, Zanetti EM, Audenino AL. Dermis mechanical behaviour after different cell removal treatments. Med Eng Phys 2016;38:862–9. 26997564 10.1016/j.medengphy.2016.02.012

[48] Rhee S. Fibroblasts in three dimensional matrices: cell migration and matrix remodeling. Exp Mol Med 2009;41:858–65. 19745603 10.3858/emm.2009.41.12.096 PMC2802681

[49] Li P , Jin D, Dou J, Wang L, Wang Y, Jin X, Han X, Kang IK, Yuan J, Shen J, Yin M. Nitric oxide-releasing poly(ε-caprolactone)/S-nitrosylated keratin biocomposite scaffolds for potential small-diameter vascular grafts. Int J Biol Macromol 2021;189:516–27. 34450147 10.1016/j.ijbiomac.2021.08.147

[50] Stoetzel S , Malhan D, Wild U, Helbing C, Hassan F, Attia S, Jandt KD, Heiss C, El Khassawna T. Osteocytes influence on bone matrix integrity affects biomechanical competence at Bone-Implant interface of bioactive-coated titanium implants in rat tibiae. Int J Mol Sci 2021;23:374. 35008800 10.3390/ijms23010374 PMC8745552

[51] Wang X , Xu S, Zhou S, Xu W, Leary M, Choong P, Qian M, Brandt M, Xie YM. Topological design and additive manufacturing of porous metals for bone scaffolds and orthopaedic implants: a review. Biomaterials 2016;83:127–41. 26773669 10.1016/j.biomaterials.2016.01.012

[52] Bakhtiary S , Chegeni A, Babaeipour V, Omidi M, Keshel SH, Khodamoradi N. Culture and maintenance of neural progressive cells on cellulose acetate/graphene-gold nanocomposites. Int J Biol Macromol 2022;210:63–75. 35537583 10.1016/j.ijbiomac.2022.05.026

[53] Qin Y , Liu A, Guo H, Shen Y, Wen P, Lin H, Xia D, Voshage M, Tian Y, Zheng Y. Additive manufacturing of Zn-Mg alloy porous scaffolds with enhanced osseointegration: in vitro and in vivo studies. Acta Biomater 2022;145:403–415. 35381400 10.1016/j.actbio.2022.03.055

[54] Porzionato A , Stocco E, Barbon S, Grandi F, Macchi V, De Caro R. Tissue-engineered grafts from human decellularized extracellular matrices: a systematic review and future perspectives. Int J Mol Sci 2018;19:4117. 10.3390/ijms19124117 PMC6321114 30567407

[55] Jakus AE , Laronda MM, Rashedi AS, Robinson CM, Lee C, Jordan SW, Orwig KE, Woodruff TK, Shah RN. “Tissue papers” from Organ-Specific decellularized extracellular matrices. Adv Funct Mater 2017;27:1700992. 29104526 10.1002/adfm.201700992 PMC5665058

[56] Wu Q , Li Y, Yang Z, Li L, Yang J, Zhu X, Liu Y, Bao J, Bu H. Ectopic expansion and vascularization of engineered hepatic tissue based on heparinized acellular liver matrix and mesenchymal stromal cell spheroids. Acta Biomater 2022;137:79–91. 34678485 10.1016/j.actbio.2021.10.017

[57] Zhang Y , Zeng Y, Xin G, Zou L, Ding Y, Duyin J. Biological function evaluation and effects of laser micro-pore burn-denatured acellular dermal matrix. Burns 2018;44:350–8. 28823469 10.1016/j.burns.2017.07.009

