# Supplementary material for: Frozen bean curd-inspired xenogeneic acellular dermal matrix with triple pretreatment approach of freeze–thaw, laser drilling and ADSCs pre-culture for promoting early vascularization and integration
Source: Regen Biomater. 2022 Aug 4;9:rbac053. doi: 10.1093/rb/rbac053 (PMC9375572; doi:10.1093/rb/rbac053)
Supplement: rbac053_Supplementary_Data [file rbac053_supplementary_data.zip › Supplementary Materials.docx]

**Supplementary Materials**

**Methods**

1.1 Swelling test

The fluid uptake ability of the PADM, FADM, LADM and FLADM was evaluated by a swelling test. Dry materials (1.0×1.5 cm^2^) were weighted (W_1_) and then immersed into PBS solution. After incubation at 37℃ for 24h, the materials were taken out from PBS, and the surface water was immediately removed by a filter paper^[1]^. The swollen samples were weighed (W_2_) and their swelling ratios were calculated using the following equation:

Swelling ratio (%) =$\frac{W2-W1}{W1}\times100$

1.2 Degradation test

Degradation test was applied to assess the degradation rates of the PADM, FADM, LADM and FLADM using collagenase solution (Collagenase NB4 Standard Grade, Nordmark, Germany). Above four dry scaffolds were cut into 6mm diameter circles and weighted (W_0_). Then they were immersed in collagenase Tris-HCL buffer solution (2U/mL), and were incubated at 37℃ with stirring for 1,3 days, respectively (n=3 per group). After being washed three times with PBS, the samples were lyophilized and weighted (W_d_)^[2, 3]^. The degradation rats of the four kinds of scaffolds were calculated using the following equation:

Degradation rate (%) = $\frac{W0-Wd}{W0}\times100$

1.3 Morphology of HUVECs on LADM and FLADM

HUVECs (5×10^4^) seeded on LADM and FLADM in size of the wells of a 24-well plate and cultured for 2 days were examined by SEM. The samples were fixed overnight at 4℃ in 2.5% glutaraldehyde and rinsed with PBS three times (15 minutes each time). Then the samples were dehydrated through a graded series of ethanol (30-100%, V/V) and dried by a CO2 critical point dryer. Once dried, the samples were observed under an SEM (ZEISS Gemini 300, Germany).

1.4 Surface morphology of LADM and FLADM

The LADM and FLADM were dried using the freezing-drier (YB-FD-1, SHYB Co., Ltd., China). The surface morphology of the dermal surface was scanned by SEM (ZEISS Gemini 300, Germany).

1.5 Adhesion of hADSCs on PADM, FADM, LADM and FLADM

hADSCs (5×10^4^) were seeded on PADM, FADM, LADM and FLADM in size of the wells of a 24-well plate and cultured for 2 days. The adhesion morphology of hADSCs on materials was observed using SEM as mentioned in section 1.3. The adhering hADSCs were also stained by TRITC-tagged-phalloidin according to the manufacturer’s instruction (Yeasen Biotechnology, China). The F-action was combined with phalloidin to exhibit cell morphology in red while the cell nucleus was stained with DAPI (SouthernBiotech, USA) in blue.

**Results and discussion**

2.1 Analysis of physical properties

The swelling ratios of FADM, LADM, and FLADM were significantly higher than that of PADM (Figure S1-A), indicating that the fluid absorption capability increased with porosity improvement. The greater the swelling ratio of the scaffold, the better nutrients are absorbed for adhering cell consumption^[4]^. The degradation results of the four groups were shown in Figure S1-B. Both on 1d and 3d, the PADM degraded most slowly, while FLADM was in the highest degradation speed. The degradation rate rises with structural porosity increasing caused by the enlarged surface^[5, 6]^.


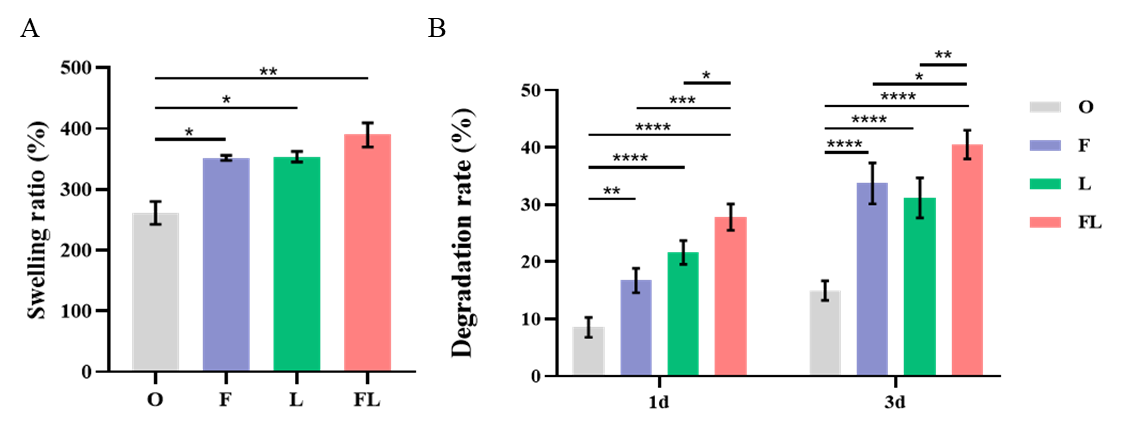


Figure S1 Physical properties. (A) Swelling ratios of PADM, FADM, LADM and FLADM. (n=3 for each group). (B) Degradation rates of PADM, FADM, LADM and FLADM after collagenase treatment for 1, 3 days. (n=3 for each group). **P<0.05, **P<0.01, ***P<0.001, ****P<0.0001.*

2.2 Adhesion of HUVECs on LADM and FLADM

As shown in Figure S2, the HUVECs adhered to the surface of LADM (group L) and FLADM (group FL) with extended pseudopodia in cluster distribution.


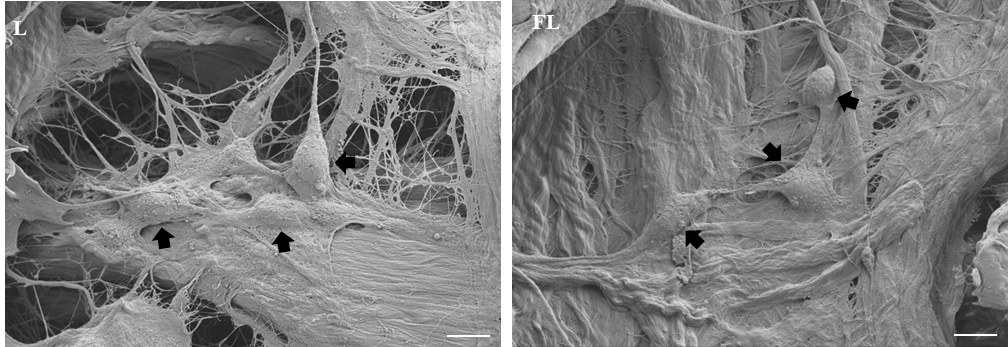


Figure S2 The SEM images of HUVECs adhesion on LADM and FLADM (the black arrows indicate the cells), bar=10μm.

2.3 Adhesion of hADSCs on PADM, FADM, LADM, and FLADM

The pores in LADM (group L) and FLADM (group FL) were introduced by laser drilling technology. The advanced technology didn’t cause evident heat damage to the surrounding tissue around the pores under the acuate parameter, which was supported by the Shanghai Institute of laser technology. As shown in Figure S3-A, the round pores were evident, and the microstructure of surrounding tissues remained. Thus, Laser drilling only changed the gross structure by mechanical force without microstructure changes. The adhering morphology of hADSCs in 4 groups was observed using SEM (Figure S3-B) and TRITC-tagged-phalloidin staining (Figure S3-C). Those adhering hADSCs exhibited spindle-like shape, elongated and extended to poles.


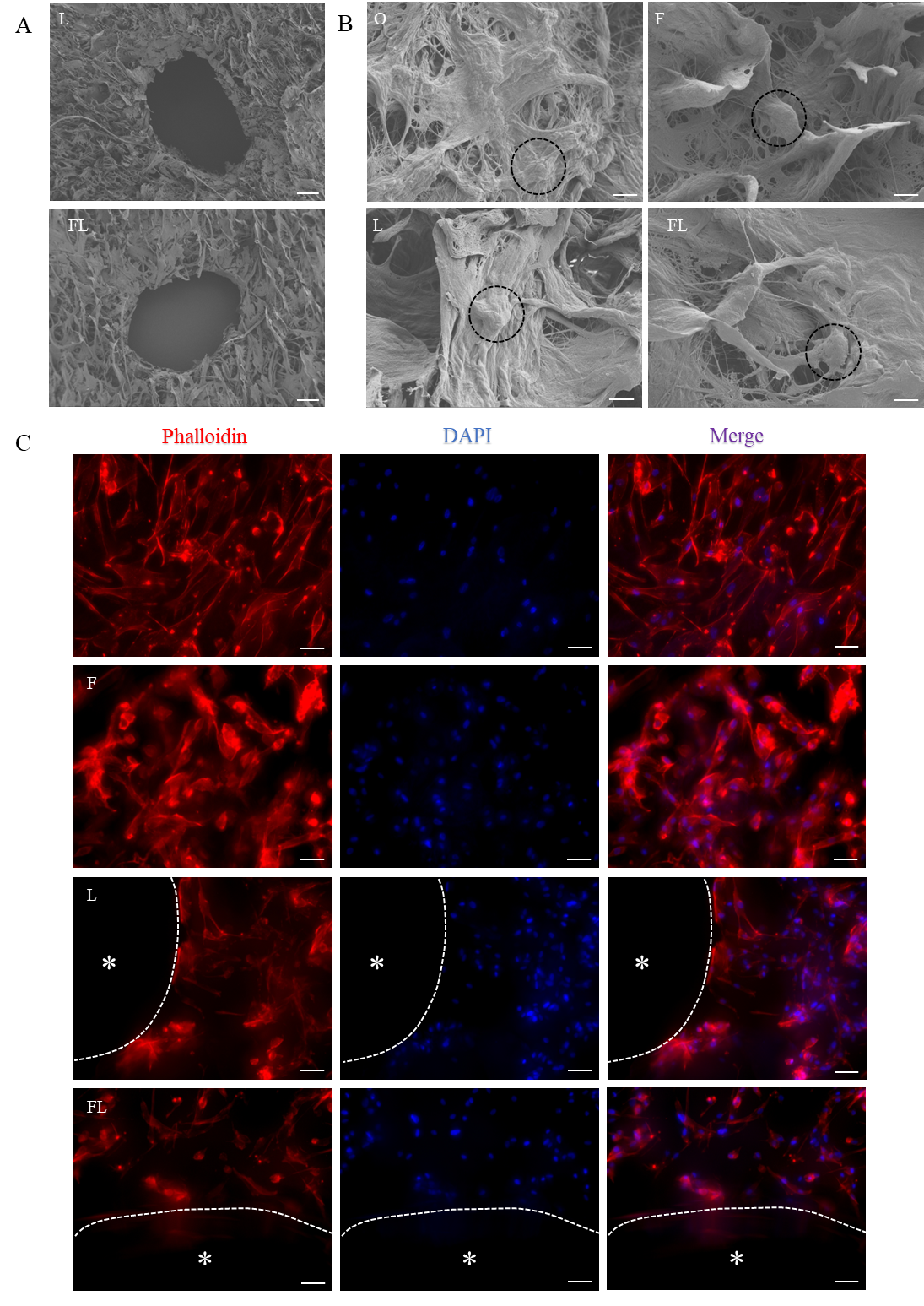


Figure S3 hADSCs adhesion on scaffolds. (A) The SEM images of the dermal surface of LADM and FLADM, bar= 200μm. (B) The SEM images of hADSCs adhering to the dermal surface of PADM, FADM, LADM and FLADM (the black circle indicates cell), bar=10μm. (C) hADSCs adhesion to PADM, FADM, LADM and FLADM stained by phalloidin (the M character indicates the pore introduced by laser, red for cytoskeleton, blue for nucleus), bar=50μm.

**Reference**

1. Amirsadeghi A, Khorram M, Hashemi SS. Preparation of multilayer electrospun nanofibrous scaffolds containing soluble eggshell membrane as potential dermal substitute. *J Biomed Mater Res A* 2021;109:1812-1827.

2. Hu Y, Liu L, Dan W, Dan N, Gu Z, Yu X. Synergistic effect of carbodiimide and dehydrothermal crosslinking on acellular dermal matrix. *Int J Biol Macromol* 2013;55:221-30.

3. Qiu J, Li J, Wang G, Zheng L, Ren N, Liu H, Tang W, Jiang H, Wang Y. In vitro investigation on the biodegradability and biocompatibility of genipin cross-linked porcine acellular dermal matrix with intrinsic fluorescence. *ACS Appl Mater Interfaces* 2013;5:344-50.

4. Bakhtiary S, Chegeni A, Babaeipour V, Omidi M, Keshel SH, Khodamoradi N. Culture and maintenance of neural progressive cells on cellulose acetate/graphene‑gold nanocomposites. *Int J Biol Macromol* 2022;210:63-75.

5. Qin Y, Liu A, Guo H, Shen Y, Wen P, Lin H, Xia D, Voshage M, Tian Y, Zheng Y. Additive manufacturing of Zn-Mg alloy porous scaffolds with enhanced osseointegration: In vitro and in vivo studies. *Acta Biomater* 2022;145:403-415.

6. Sousa AM, Amaro AM, Piedade AP. 3D Printing of Polymeric Bioresorbable Stents: A Strategy to Improve Both Cellular Compatibility and Mechanical Properties. *Polymers (Basel)* 2022;14.
