# Supplementary material for: Frozen bean curd-inspired xenogeneic acellular dermal matrix with triple pretreatment approach of freeze–thaw, laser drilling and ADSCs pre-culture for promoting early vascularization and integration
Source: Regen Biomater. 2022 Aug 4;9:rbac053. doi: 10.1093/rb/rbac053 (PMC9375572; doi:10.1093/rb/rbac053)
Supplement: rbac053_Supplementary_Data [file rbac053_supplementary_data.zip › Point to point response.docx]

July, 2022

Dear Dr. Nicholas Peppas

Editor in chief

Regenerative Biomaterials

On behalf of all the contributing authors, I would like to express our sincere appreciation for your letter and the constructive comments by the referees regarding our manuscript, entitled “Frozen bean curd-inspired Xenogeneic acellular dermal matrix with triple pretreatment approach of freeze-thaw, laser drilling and ADSCs pre-culture for promoting early vascularization and integration”.

Based on the comments and suggestions, we have made corresponding modifications to our manuscript. In the revised version, all the changes have been highlighted. **All changes in the text have been highlighted in red while the changes that correspond to the question of reviewers are in blue.** Point-by-point responses to the comments are provided in this letter.

Once again, we thank you for your support and help. We would be open to making further changes to improve the paper and to facilitate publication in your journal.

**Response to Editorial Office**

Comments:

**Editorial Office:**

1)The size of the graphic abstract does not meet the requirement we demand. Please prepared a graphic abstract in the actual size (1.4 in. high and 3.3 in. wide, or 3.6 cm ×8.4 cm) and show it immediately following the abstract and do not enlarge or reduce it in your revised manuscript.

2)according to our Authors' Guidelines: for references to journal articles, all authors should be included.

**Response:** Thank you for your comments and valuable suggestions for my manuscript.

1. We apologize for our negligence in the actual size of the graphic abstract. We have modified the final size in accordance with 3.6cm high and 8.4cm wide displayed as follows, and embedded the illustration immediately following the abstract with the actual size in our revised manuscript.

Figure Graphic abstract.

2. We apologize for our thoughtlessness in reference format. We have modified the reference format according to the journal’s requirement in the revised manuscript. For references to the journal articles, all authors have been listed and marked in **blue**.

**Reviewer 1:**

In this manuscript, the authors presented an interesting approach to the fabrication of an AMD into a three-dimensional porous scaffold seeded with hADSCs by utilizing freeze-thaw, laser drilling and hADSCs pre-culture. However, some format modification and data supplement need to be provided.

1) The cell morphology of ADSCs on the scaffolds should be tested by SEM to further verify the biocompatibility.

2) The characterization of FLAMD scaffold is insufficiently explored. The author only tested the macrostructure of the FLAMD scaffold. Whether the microstructure of FLAMD scaffold changed after treated by laser drilling? Therefore, SEM to confirm the microstructure is suggested.

3) In this research, the mechanical properties of LADM and FLADM was increased after implanted in vivo after 4w. The author explained as “which may be related to the degradation of the materials and the matrix secretion to remodel materials by infiltrating cells simultaneously”, which may need more evidence. Degradation test in different microenvironment, swelling test or other methods are suggested.

4) In summary, the angiogenesis ability of the 4 groups has no significant difference, which may prove that changing the porosity of the AMD materials may not improve the angiogenesis ability?

5) In the histology images, the scaffold remnants must be marked.

6) Please confirm the accuracy of the grammar, spelling, punctuation, and verb tenses again to make the manuscript more understandable.

7) The recently published review and research articles should be cited and discussed in the revision.

**Response**: Thank you for your valuable suggestion about our work. The paper was revised and polished by an English native to correct the errors of grammar, spelling, punctuation and verb tenses for more clear expression before the revised edition submission. The date of supplementary tests according to your suggestion has also been provide to support the opinions in this article. All the changes in the text are shown in **red** while contents related to the reviewers’ questions are shown in **blue** in the revised manuscript. The details are as follows.

**Q1:** **The cell morphology of ADSCs on the scaffolds should be tested by SEM to further verify the biocompatibility.**

A: Thank you for your instructive idea. We have supplemented the cell morphology of ADSCs on the scaffold observed by SEM in the supplementary document. Additionally, the adhesion morphology of hADSCs was also observed using phalloidin that was specially combined with F-action. The corresponding experimental methods and results were put in the supplementary document.

The supplementary text: hADSCs (5×10^4^) were seeded on PADM, FADM, LADM, and FLADM in size of the wells of a 24-well plate and cultured for 2 days. The morphology of adhered hADSCs on materials was observed using SEM as mentioned. The adhering hADSCs were also stained by TRITC-tagged-phalloidin according to the manufacturer’s instruction (Yeasen Biotechnology, China). The F-action was combined with phalloidin to exhibit cell morphology in red while the cell nucleus was stained with DAPI (SouthernBiotech, USA) in blue.

The adhering morphology of hADSCs in 4 groups was observed using SEM (Figure A) and TRITC-tagged-phalloidin staining (Figure B). Those adhering hADSCs exhibited spindle-like shape, elongated and extended to poles.

Figure 1 hADSCs adhesion on scaffolds. (A) The SEM images of hADSCs adhering to the dermal surface of PADM, FADM, LADM and FLADM (the black circle indicates cell), bar=10μm. (B) hADSCs adhesion to PADM, FADM, LADM and FLADM stained by phalloidin (the M character indicates pores introduced by laser, red for cytoskeleton, blue for nucleus), bar=50μm.

**Q2: The characterization of FLAMD scaffold is insufficiently explored. The author only tested the macrostructure of the FLAMD scaffold. Whether the microstructure of FLAMD scaffold changed after treated by laser drilling? Therefore, SEM to confirm the microstructure is suggested.**

A: Thank you for your professional instruction. We apologize for the missing SEM images of microstructure of FLADM. The advanced laser drilling technique didn’t cause evident heat damage to the surrounding tissue around the pores under the arcuate parameter, which was supported by the Shanghai Institute of laser technology. Laser drilling only changed the gross structure by mechanical force without microstructure changes. The SEM images of LADM and FLADM were shown as follows. Thus, we were reasonable to believe that microstructure of FLADM remained its original morphology. The SEM images have been put in the supplementary materials.

The supplementary text: The LADM and FLADM were dried using the freezing-drier (YB-FD-1, SHYB Co., Ltd., China). The surface morphology of the dermal surface was scanned by SEM (ZEISS Gemini 300, Germany).

The pores in LADM (group L) and FLADM (group FL) were introduced by laser drilling technology. The advanced technology didn’t cause evident heat damage to the surrounding tissue around the pores under the acuate parameter, which was supported by the Shanghai Institute of laser technology. As shown in Figure 2, the round pores were evident, and the microstructure of surrounding tissues remained. Thus, Laser drilling only changed the gross structure by mechanical force without microstructure changes.

Figure 2 The SEM images of the dermal surface of LADM and FLADM, bar= 200μm.

**Q3: In this research, the mechanical properties of LADM and FLADM was increased after implanted in vivo after 4w. The author explained as “which may be related to the degradation of the materials and the matrix secretion to remodel materials by infiltrating cells simultaneously”, which may need more evidence. Degradation test in different microenvironment, swelling test or other methods are suggested.**

A: Thank you for your meaningful advisement. Based on our previous research and published articles^[1, 2]^ , it has been found that the implanted ADM gradually degraded in vivo and was replaced by newly-born collagen tissue. It could be one important reason to explain the mechanical changing^[3]^. A similar phenomenon has been described in other biomaterials as well. Risa Ikeda etc. described that the PLG scaffolds for osteochondral defects showed better mechanical properties after implantation^[4]^. He Wang etc. also reported that the composite hydrogel presented better mechanical property in vivo ^[5]^.

In addition, biomaterials with high porosity improved their biological process^[6]^. Chao Wang etc. found that the Ti6Al4V scaffold for bone defects with higher porosity exhibited better mechanical property after 12w scaffold implantation due to convenient tissue ingrowth and blood vessel formation^[7]^. Xiaojun Wang etc. also described that the mechanical properties of the biomaterials for repairing pelvic floor dysfunction on post-operative 90 days were all better than those on 30 days due to integration^[8]^. Thus, We are reasonable to believe the LADM and FLADM presented a mechanical increase at 4w relating to their higher porosity and enhanced remolding process.

The degradation rate rises with the structural porosity increasing caused by the enlarged surface ^[9, 10]^. In addition, the degradation behavior of the biomaterials is closely related to the microenvironments ^[11]^. In order to further explain the mechanical properties changing, the degradation test using the collagenase solution and swelling test were applied to PADM, FADM, LADM, and FLADM.

The fluid uptake ability of the PADM, FADM, LADM and FLADM was evaluated by a swelling test. Dry materials (1×1.5 cm^2^) were weighted (W_1_) and then immersed into PBS solution. After incubation at 37℃ for 24h, the materials were taken out from PBS, and the surface water was immediately removed by a filter paper^[12]^. The swollen samples were weighed (W_2_) and their swelling ratios were calculated using the following equation:

Swelling ratio (%) =$\frac{W2-W1}{W1}\times100$

A degradation test was applied to assess the degradation rates of the PADM, FADM, LADM and FLADM using collagenase solution (Collagenase NB4 Standard Grade, Nordmark, Germany). Above four dry scaffolds were cut into 6mm diameter circles and weighted (W_0_). Then they were immersed in collagenase Tris-HCL buffer solution (2U/mL), and were incubated at 37℃ with stirring for 1,3 days, respectively (n=3 per group). After being washed three times with PBS, the samples were lyophilized and weighted (W_d_)^[13, 14]^. The degradation rats of the four kinds of scaffolds were calculated using the following equation:

Degradation rate (%) = $\frac{W0-Wd}{W0}\times100$

The swelling ratios of FADM, LADM, and FLADM were significantly higher than that of PADM (Figure 3A), indicating that the fluid absorption capability increased with porosity improvement. The greater the swelling ratio of the scaffold, the better nutrients are absorbed for adhering cell consumption^[15]^. The degradation results of the four groups were shown in Figure 3B. Both on 1d and 3d, the PADM degraded most slowly, while FLADM was in the highest degradation speed. The difference in degradation rates among the four groups was mainly caused by porosity variance. The corresponding experimental methods and results were put in the supplementary document.

Figure 3 (A) Swelling ratios of PADM, FADM, LADM and FLADM. (n=3 for each group). (B) Degradation rates of PADM, FADM, LADM and FLADM after collagenase treatment for 1, 3 days. (n=3 for each group). **P<0.05, **P<0.01, ***P<0.001, ****P<0.0001.*

Based on the above discussion, we are reasonable to believe that the mechanical properties of LADM and FLADM with higher porosity improved caused by the balance of faster degradation and better remolding process. The further explanation has been supplemented in chapter 3.5 (In vivo experiment and the changes in mechanical properties) in **blue**.

The revised text:

Degradation of the materials and the matrix deposition to remodel materials by infiltrating cells simultaneously during the regenerative process. Though the stiffness and strength of a scaffold decrease with the porosity increasing, the biological processes enhanced. Besides, the swelling ratios of FADM, LADM, and FLADM were significantly higher than that of PADM (Figure S1-A) due to porosity increase, indicating that the better nutrients are absorbed in the scaffolds for adhering cells. In addition, the degradation rate rises with structure porosity increasing because of the enlarged surface. The degradation results of the four groups were shown in Figure S1-B. Both on 1d and 3d, the PADM degraded most slowly, while FLADM was in the highest degradation speed. The difference in degradation rates among the four groups was mainly caused by porosity variance. Thus, the biomechanical properties decreased at first, and increased subsequently, indicating that there was a balance between the material degradation and extracellular matrix deposition, and the remolding process of FADM, LADM, and FLADM was enhanced due to porosity increase.

**Q4: In summary, the angiogenesis ability of the 4 groups has no significant difference, which may prove that changing the porosity of the AMD materials may not improve the angiogenesis ability?**

A: Thank you for your thought-provoking question. Though the difference among the 4 groups was unremarkable in HUVEC proliferation and VEGF secretion in vitro, the genes expression and tube formation in vitro displayed a marked difference in vitro. The physiological angiogenesis is a complicated process depending on the balance of positive and negative angiogenic modulators and requires the functional activities of other molecules like extracellular matrix proteins^[16]^. The regulating factors appear in specific spatio-temporal patterns, indicating that the above inconsistent results were accepted.

More importantly, the number of vessels and diameter of vessels of LADM and FLADM had significant differences from those on FADM and PADM at 4w in vivo. The results of in vivo experiments strongly supported the effectiveness of the pre-treatments, and the in vitro experiments were to explore its possible mechanism. As the discussion about Q3, the biomaterial with high porosity is conducive to biological processes, including the new vessel formation. Fupo He etc. described that the calcium phosphate-based bioceramic scaffolds with interconnected micropores facilitated the new vessel formation compared with those without pores at the sixth-week postimplantaion^[17]^. All in all, the porosity increasing enhanced the neovascularization ability of the ADM material.

**Q5: In the histology images, the scaffold remnants must be marked.**

A: Thank you for your good advice. We have marked the scaffold remnants in the slices stained with HE. The revised picture was shown as follows. The M characters represented the implanted material while the pentagram indicated the pore produced by laser drilling. Because the Masson staining and CD31 staining focused on the material itself, no additional auxiliary line was drawn. Figure 7 has been updated in the revised manuscript and the legend has been modified in **blue**.

Figure 4 The HE staining, bar=200μm. (the M characters represented the implanted material, and the pentagram indicated the pore produced by laser drilling)

**Q6: Please confirm the accuracy of the grammar, spelling, punctuation, and verb tenses again to make the manuscript more understandable.**

A: Thank you for your kind reminder. We have carefully looked over our article once again and corrected the errors of grammar, spelling, punctuation, and verb tenses. A senior English editor has revised and polished the paper to make it less colloquial and easier to understand. All the changes are highlighted in **red** in the revised manuscript.

**Q7: The recently published review and research articles should be cited and discussed in the revision.**

A: Thank you for your constructive suggestion. We have updated our reference list and newly added references in **red**. The main content of new references was listed in a table as follows.

| Publish year | Title | Content |
| --- | --- | --- |
| 2021 | Membranous Extracellular Matrix-Based Scaffolds for Skin Wound Healing | It is a review that concluded the common extracellular matrix-based scaffolds for skin regeneration including acellular dermal matrix. It was cited for supporting characteristics of acellular dermal matrix, like biocompatibility, abundant source, and immunological inertia. |
| 2013 | Angiogenesis | It is a typical and comprehensive chapter to illustrate an angiogenesis process and is cited. |
| 2021 | Nitric oxide-releasing poly(ε-caprolactone)/S-nitrosylated keratin biocomposite scaffolds for potential small-diameter vascular grafts. | It is an original article about a new vascular scaffold. It was cited to prove the truth of the degradation and remolding process proceeding simultaneously. |
| 2021 | Osteocytes Influence on Bone Matrix Integrity Affects Biomechanical Competence at Bone-Implant Interface of Bioactive-Coated Titanium Implants in Rat Tibiae. | The article was cited to illustrate the balance of degradation and formation during the remodeling process. |

**Reference**

1.	Li P, Jin D, Dou J, Wang L, Wang Y, Jin X, Han X, Kang IK, Yuan J, Shen J, Yin M. Nitric oxide-releasing poly(ε-caprolactone)/S-nitrosylated keratin biocomposite scaffolds for potential small-diameter vascular grafts. *Int J Biol Macromol* 2021;189:516-527.

2.	Stoetzel S, Malhan D, Wild U, Helbing C, Hassan F, Attia S, Jandt KD, Heiss C, El Khassawna T. Osteocytes Influence on Bone Matrix Integrity Affects Biomechanical Competence at Bone-Implant Interface of Bioactive-Coated Titanium Implants in Rat Tibiae. *Int J Mol Sci* 2021;23.

3.	Liu J, Li T, Zhang H, Zhao W, Qu L, Chen S, Wu S. Electrospun strong, bioactive, and bioabsorbable silk fibroin/poly (L-lactic-acid) nanoyarns for constructing advanced nanotextile tissue scaffolds. *Mater Today Bio* 2022;14:100243.

4.	Ikeda R, Fujioka H, Nagura I, Kokubu T, Toyokawa N, Inui A, Makino T, Kaneko H, Doita M, Kurosaka M. The effect of porosity and mechanical property of a synthetic polymer scaffold on repair of osteochondral defects. *Int Orthop* 2009;33:821-8.

5.	Wang H, Hu B, Li H, Feng G, Pan S, Chen Z, Li B, Song J. Biomimetic Mineralized Hydroxyapatite Nanofiber-Incorporated Methacrylated Gelatin Hydrogel with Improved Mechanical and Osteoinductive Performances for Bone Regeneration. *Int J Nanomedicine* 2022;17:1511-1529.

6.	Wang X, Xu S, Zhou S, Xu W, Leary M, Choong P, Qian M, Brandt M, Xie YM. Topological design and additive manufacturing of porous metals for bone scaffolds and orthopaedic implants: A review. *Biomaterials* 2016;83:127-41.

7.	Wang C, Xu D, Lin L, Li S, Hou W, He Y, Sheng L, Yi C, Zhang X, Li H, Li Y, Zhao W, Yu D. Large-pore-size Ti6Al4V scaffolds with different pore structures for vascularized bone regeneration. *Mater Sci Eng C Mater Biol Appl* 2021;131:112499.

8.	Wang X, Chen Y, Fan Z, Hua K. Evaluating tissue-engineered repair material for pelvic floor dysfunction: a comparison of in vivo response to meshes implanted in rats. *Int Urogynecol J* 2021.

9.	Qin Y, Liu A, Guo H, Shen Y, Wen P, Lin H, Xia D, Voshage M, Tian Y, Zheng Y. Additive manufacturing of Zn-Mg alloy porous scaffolds with enhanced osseointegration: in vitro and in vivo studies. *Acta Biomater* 2022.

10.	Sousa AM, Amaro AM, Piedade AP. 3D Printing of Polymeric Bioresorbable Stents: A Strategy to Improve Both Cellular Compatibility and Mechanical Properties. *Polymers (Basel)* 2022;14.

11.	Su Y, Fu J, Lee W, Du S, Qin YX, Zheng Y, Wang Y, Zhu D. Improved mechanical, degradation, and biological performances of Zn-Fe alloys as bioresorbable implants. *Bioact Mater* 2022;17:334-343.

12.	Amirsadeghi A, Khorram M, Hashemi SS. Preparation of multilayer electrospun nanofibrous scaffolds containing soluble eggshell membrane as potential dermal substitute. *J Biomed Mater Res A* 2021;109:1812-1827.

13.	Hu Y, Liu L, Dan W, Dan N, Gu Z, Yu X. Synergistic effect of carbodiimide and dehydrothermal crosslinking on acellular dermal matrix. *Int J Biol Macromol* 2013;55:221-30.

14.	Qiu J, Li J, Wang G, Zheng L, Ren N, Liu H, Tang W, Jiang H, Wang Y. In vitro investigation on the biodegradability and biocompatibility of genipin cross-linked porcine acellular dermal matrix with intrinsic fluorescence. *ACS Appl Mater Interfaces* 2013;5:344-50.

15.	Bakhtiary S, Chegeni A, Babaeipour V, Omidi M, Keshel SH, Khodamoradi N. Culture and maintenance of neural progressive cells on cellulose acetate/graphene‑gold nanocomposites. *Int J Biol Macromol* 2022;210:63-75.

16.	Ribatti D. Angiogenesis. In: Maloy S, Hughes K (eds). *Brenner's Encyclopedia of Genetics (Second Edition)*. San Diego: Academic Press, 2013, 130-132.

17.	He F, Lu T, Fang X, Feng S, Feng S, Tian Y, Li Y, Zuo F, Deng X, Ye J. Novel Extrusion-Microdrilling Approach to Fabricate Calcium Phosphate-Based Bioceramic Scaffolds Enabling Fast Bone Regeneration. *ACS Appl Mater Interfaces* 2020;12:32340-32351.

**Reviewer 2:**

This paper reported a kind of Xenogeneic acellular dermal matrix (ADM) which was inspired by the manufacturing procedure of frozen bean curd and combined freeze-thaw with laser drilling and pre-culture with ADSC as a wound dressing for the treatment of wounds. In this study, the ADM with triple pretreatment can optimize the internal structure of ADM, and may yield clinical significance by promoting early cell infiltration, vascularization, and integration with surrounding tissues. Though the topic is novel, the integrality and logicality even some details of this paper should be improved. The following comments and suggestions should be helpful for the improvement of the paper.

1) In present study, freeze-thaw combined laser drilling was used to enhance the porosity of porcine acellular dermal matrixs, which provide excellent microenvironment for exogenous cells. The SEM Characterization of LADM and FADM and proliferation and angiogenesis of HUVECs on LADM and FADM should be presented in Fig.2 and Fig.3.

2) In Fig.8, at 4w, the blood vessel diameters on group L and group FL were not 10-15μm according the bar.

3) Line spacing and character spacing are not standard, some places large and some places small.

4) According to Fig.3F, there is no significant difference between group O and group F, so what is the proof to prove HUVECs on FADM proliferate faster and adhere more?

5) The expressions of VEGF in group F, group L and group FL were significantly higher than that in group O. Why there was no statistical difference of VEGF in Fig.5B?

6) The result is described as IGF-1, but in the figure is labeled as IGF.

**Response:** Thank you for your meticulous review and inspiring comments on our work. We apologize for mistakes in word format and figure labels. We have modified the figure and format according to relevant requirements in the revised manuscript and rewritten the contents that are not clearly expressed. All the changes in the text are shown in **red** while contents related to the reviewers’ questions are shown in **blue** in the revised manuscript. The details are as follows.

**Q1 In present study, freeze-thaw combined laser drilling was used to enhance the porosity of porcine acellular dermal matrixs, which provide excellent microenvironment for exogenous cells. The SEM Characterization of LADM and FADM and proliferation and angiogenesis of HUVECs on LADM and FADM should be presented in Fig.2 and Fig.3.**

A: Thank you for your valuable suggestion. We apologize for the insufficient SEM images of LADM and FLADM groups. We have supplemented the above-mentioned images. Figure 2 showed the physicochemical properties of the PADM and FADM to discuss the porosity change and illustrate the effectiveness of freeze-thaw treatment. Figure 3 displayed the HUVECS behaviors on the PADM and FADM to verify the pro-angiogenic effect of freeze-thaw treatment. Thus, we thought that it was suitable to supplement the images in the supplementary document.

The supplementary text: The LADM and FLADM were dried using the freezing-drier (YB-FD-1, SHYB Co., Ltd., China). The surface morphology of the dermal surface was scanned by SEM (ZEISS Gemini 300, Germany). HUVECs (5×10^4^) seeded on LADM and FLADM in size of the wells of a 24-well plate and cultured for 2 days were examined by SEM. The samples were fixed overnight at 4℃ in 2.5% glutaraldehyde and rinsed with PBS three times (15 minutes each time). Then the samples were dehydrated through a graded series of ethanol (30-100%, V/V) and dried by a CO2 critical point dryer. Once dried, the samples were observed under an SEM (ZEISS Gemini 300, Germany).

The pores in LADM (group L) and FLADM (group FL) were introduced by advanced laser drilling technology without heat damage to surrounding tissues around the pores (Figure A). Under the acuate parameter, Laser drilling only changed the gross structure by mechanical force without microstructure changes. HUVECs adhered to the surface of LADM (group L) and FLADM (group FL) with extended pseudopodia in cluster distribution.

Figure 1 (A) The SEM images of the dermal surface of LADM and FLADM, bar= 200μm. (B) The SEM images of HUVECs adhesion on LADM and FLADM (the black arrows indicate the cells), bar=10μm.

**Q2 In Fig.8, at 4w, the blood vessel diameters on group L and group FL were not 10-15μm according the bar.**

A: Thank you for your question. As the enlarged figure equal to the ratio of the original picture displayed as follows, the blood vessel diameters on group L and group FL at 4w were all in the range of 10-15μm. The original date shown in table format was attached as well. We ensured that the uploaded picture was clear enough to identify. Thank you for your careful review on our manuscript once again.

Figure 2 The diameter of blood vessels (n=3 for each group). **P<0.05, **P<0.01, ***P<0.001, ****P<0.0001.*

Figure 3 Diameter measurement of new blood vessels using Image J software (the LADM group on 4w).

Table 1 The diameter of the blood vessels

| **Group**  **Time** | **L/μm** | | | **FL/μm** | | |
| --- | --- | --- | --- | --- | --- | --- |
| **2w** | 6.83 | 6.52 | 6.95 | 7.20 | 7.01 | 6.14 |
| **4w** | 11.00 | 14.53 | 11.66 | 11.83 | 15.62 | 11.10 |

**Q3 Line spacing and character spacing are not standard, some places large and some places small.**

A: Thank you for your kind reminder. We apologize for our carelessness on word format, especially on line spacing and character spacing. We have unified the standard of line spacing and character spacing according to the requirements of the journal on our revised manuscript.

**Q4 According to Fig.3F, there is no significant difference between group O and group F, so what is the proof to prove HUVECs on FADM proliferate faster and adhere more?**

A: Thank you for your question. We apologize for the ambiguous statement in our article. Though the difference was not statistically significant, it still reflected a trend between the two groups. There were mean differences in live cell numbers and OD values between group O and group F. For visualizing the difference, the descriptive results of live cell numbers and OD values, including mean and value range, were shown in the following picture. No matter the live cell number or the OD values, the mean of group F was better than group O. In order to accurately describe the phenomenon without ambiguity, we modified the statement in **blue** in our revised manuscript.

Figure 4 The descriptive results of live cell numbers (A) and OD values (B). (The line indicates the mean; the upper and lower borders of the box represent the maximum and minimum respectively.)

The revised text: Therefore, HUVECs on FADM presented a trend of faster proliferation and more adhesion.

**Q5 The expressions of VEGF in group F, group L and group FL were significantly higher than that in group O. Why there was no statistical difference of VEGF in Fig.5B?**

A: Thank you for your question. Gene expression is a two-step process including transcription and translation. qPCR reflects the comparative expression of mRNA while Elisa tests the protein content. The problems, like mRNA process and degradation, post-translation modification would interfere the target protein production. In addition, the sensitivity of the experiments is varied, PCR illustrates the difference in copy numbers (2^n^) while Elisa expresses the changes in concentration (pg/ml). In addition, the VEGF family has a complex organization of the VEGF gene and multiple characteristics of the VEGF protein. Taking the VEGF-A as an example, the precursor mRNA from VEGF-A transcription could translate into different VEGF-A proteins with varied fragments through alternative splicing, including VEGF121, VEGF165, VEGF 189, and VEGF 206. VEGF 121 is a freely diffusible protein; VEGF 165 is also a secreted protein, but a significant fraction remains bound to the cell surface and the extracellular matrix (ECM). In contrast, VEGF 189 and VEGF206 are almost completely sequestered in the ECM. However, the Elisa results merely reflects the floating VEGF proteins^[1]^.

Thus, the PCR results of VEGF is not always consistent to those using Elisa.

The revised text: It may be caused by the complex mechanism of gene expression and protein translation of VEGF. Part of VEGF proteins reminds bound to the extracellular matrix resulting in inconsistent outcomes with the PCR results.

**Q6 The result is described as IGF-1, but in the figure is labeled as IGF.**

A: Thank you for your correction. The IGF-1 and IGF were the same gene in this article, we have corrected the figure 5 label on the revised manuscript.

**Reference**

1.	Ferrara N. Vascular endothelial growth factor: molecular and biological aspects. *Curr Top Microbiol Immunol* 1999;237:1-30.
